# Long-term protective efficacy with a BCG-prime ID93/GLA-SE boost regimen against the hyper-virulent *Mycobacterium tuberculosis* strain K in a mouse model

**DOI:** 10.1038/s41598-019-52146-0

**Published:** 2019-10-29

**Authors:** Kee Woong Kwon, Ara Lee, Sasha E. Larsen, Susan L. Baldwin, Rhea N. Coler, Steven G. Reed, Sang-Nae Cho, Sang-Jun Ha, Sung Jae Shin

**Affiliations:** 1 Department of Microbiology, Institute for Immunology and Immunological Disease, Brain Korea 21 PLUS Project for Medical Science, Yonsei University College of Medicine, Seoul, 03722 South Korea; 2 Department of Biochemistry, College of Life Science & Biotechnology, Yonsei University, Seoul, 03722 South Korea; 3 Infectious Disease Research Institute, 1616 Eastlake Ave E, Suite 400, Seattle, WA 98102 USA; 4 Department of Global Health, University of Washington, Seattle, USA; 5 PAI Life Sciences Inc., Seattle, USA

**Keywords:** Tuberculosis, Protein vaccines

## Abstract

Since ID93/GLA-SE was developed as a targeted BCG-prime booster vaccine, in the present study, we evaluated the protective efficacy of ID93/GLA-SE as a boost to a BCG-prime against the hypervirulent *Mycobacterium tuberculosis* (Mtb) K challenge to provide further information on the development and application of this vaccine candidate. Boosting BCG with the ID93/GLA-SE vaccine significantly reduced bacterial burden at 16 weeks post-challenge while the BCG vaccine alone did not confer significant protection against Mtb K. The pathological analysis of the lung from the challenged mice also showed the remarkably protective boosting effect of ID93/GLA-SE on BCG-immunised animals. Moreover, qualitative and quantitative analysis of the immune responses following ID93/GLA-SE-immunisation demonstrated that ID93/GLA-SE was able to elicit robust and sustained Th1-biased antigen-specific multifunctional CD4^+^ T-cell responses up to 16 weeks post-challenge as well as a high magnitude of an antigen-specific IgG response. Our findings demonstrate that the ID93/GLA-SE vaccine candidate given as a BCG-prime boost regimen confers a high level of long-term protection against the hypervirulent Mtb Beijing infection. These findings will provide further and more feasible validation for the potential utility of this vaccine candidate particularly in East-Asian countries, with the predominance of the Beijing genotype, after BCG vaccination.

## Introduction

Tuberculosis (TB), caused by *Mycobacterium tuberculosis* (Mtb), is the number one infectious disease causing human death in the world. In 2017, TB ranked as one of the top ten killers, causing an estimated 10 million new cases with 1.6 million people having died of TB disease. In addition, approximately 1.7 billion people, equivalent to roughly 23% of the global population, are estimated to harbor a latent TB infection (LTBI) and therefore carry the risk of progressing to active TB disease during their lifetime. The global emergence and spread of Mtb strains resistant to one or more front-line TB drugs also contributes to the challenges of treating these burdensome infections^1^.

The WHO End TB Strategy, has put forth priorities for Mtb control with the ambitious target of reducing global TB disease burden. However, subsequent reporting on TB disease trends are to date inadequate to meet these goals^1^. Among important TB control measures, the development of novel more effective TB vaccines is one such measure urgently needed to archive this goal.

In 2018, the clinical efficacy trials of two TB multi-antigenic subunit vaccines have demonstrated promising results and have helped to advance the experimental design strategies in the TB vaccine field and candidate pipeline. First, the H4:IC31 candidate was evaluated as a prevention of infection (prophylactic) strategy in high-transmission risk adolescents^2^. In this trial, the H4:IC31 vaccination reduced the rate of sustained QuantiFERON-TB Gold In-tube assay conversion, which may reflect sustained Mtb infection, with an efficacy of 30.5% along with no serious adverse events. Second, the M72/AS01_E_ vaccine candidate was evaluated as a prevention of TB disease trial in Mtb-infected, healthy adults^3^.

Interestingly, the two subunit vaccine candidates mentioned above have three common properties; (1) multi-antigenic protein vaccine produced as a single fusion protein, (2) formulated in their own unique adjuvants, and (3) evaluated in *Mycobacterium bovis* Bacillus Calmette–Guérin (BCG)-vaccinated healthy populations in TB endemic regions, mainly in South African countries. In addition, as with most vaccine candidates, the two subunit vaccines were evaluated and optimised in a variety of animal models prior to advancing into clinical trials^4–9^. Moreover, both vaccine candidates effectively boosted a BCG-induced immune response with the maintenance of a long-term protection and persistent Th1-biased multifunctional CD4^+^ T-cell responses in preclinical TB models^4,9^.

Likewise, novel subunit vaccine candidate ID93, which has similar properties as the two above-mentioned vaccine candidates including a multi-antigenic fusion protein combined with a synthetic TLR4 glucopyranosyl lipid adjuvant formulated in a stable oil-in-water emulsion (GLA-SE), has entered into clinical trials. Recently reported from a phase 1 clinical trial, an acceptable safety profile and durable Th1-immunogenicity response was attributed to ID93/GLA-SE given to previously BCG-vaccinated healthy adults^10^.

Previously, our group demonstrated that vaccination with the ID93/GLA-SE candidate induces a robust reduction of bacterial burden against challenge with the hypervirulent Mtb K/Beijing clinical isolate from a TB outbreak in high schools of South Korea^11^. Protection with this vaccine was characterised by pulmonary Th1-polarised T-cell immune responses in head-to-head comparison between BCG and the ID93/GLA-SE vaccine in a standard mouse model^12^. In a separate manuscript, the ID93/GLA-SE vaccine was shown to elicit protection against Mtb W/Beijing HN878 and provide long-lived vaccine-specific Th1-type T-cell immunity in a standard mouse model^13^, which also included BCG vaccination as a comparator. These findings support the potential use of this vaccine candidate specifically in East-Asian countries where Beijing family strains are predominant^14^. In addition, the Mtb Beijing lineage strains are expanding globally with the massive spread of multidrug-resistant TB more rapidly than other lineages^15^, indicating that evaluation of vaccine candidates against the Beijing family in relevant preclinical models is urgently required and may significantly impact global health^16^. Thus, both investigations strengthen the rationale for evaluating the long-lived efficacy of subunit TB vaccine candidates, like ID93/GLA-SE, against a clinically relevant hypervirulent Mtb isolate in an accessible preclinical animal model.

BCG, the only licensed vaccine against TB, is given to neonates or young children in many countries even though variable and waning efficacy of protection against pulmonary TB has been observed across several clinical studies^17,18^. Nevertheless, recent clinical meta-analyses studies have demonstrated that individuals who are relatively naïve to mycobacterial exposure prior to BCG vaccination are associated withenhanced efficacy endpoints^19^, and significant BCG-induced protection against severe disseminated Mtb infection supports its use^20^. In addition, two promising multi-antigenic subunit vaccine candidates, H4:IC31 and M72/AS01_E_, mentioned above^2,3^, were evaluated as a BCG booster vaccine in clinical trials. Thus, although BCG is unable to eradicate TB disease when given alone, we propose that leveraging BCG in a prime-boost regimen may provide a promising vaccine strategy for the prevention of TB disease.

The capacity of T-cells to be expanded and differentiated into effector T-cells from central memory T-cells in response to BCG stimulation wanes with increased age in healthy children, suggesting that booster vaccines should be considered to maintain the antigen-specific T-cells and possibly enhance the duration of protection afforded by BCG^21^. For these reasons, in the current study, we evaluated protective efficacy of ID93/GLA-SE as a BCG-prime booster vaccine against Mtb K to provide further and more feasible validation.

Although ID93/GLA-SE TB vaccine candidate is currently under evaluation in human clinical trials and has been tested in numerous animal models^22–24^ to our knowledge, evaluation of this TB vaccine as a boost to a BCG-prime in a long-term standard mouse TB model has not been previously reported. For these reasons, in the current study, we evaluated protective efficacy of ID93/GLA-SE as a BCG-prime booster vaccine against Mtb K in a murine model to provide further and more feasible validation of this subunit vaccine candidate.

## Results

### Antigen-specific immune responses following ID93/GLA-SE immunisation in BCG-vaccinated mice

**Figure 1:**
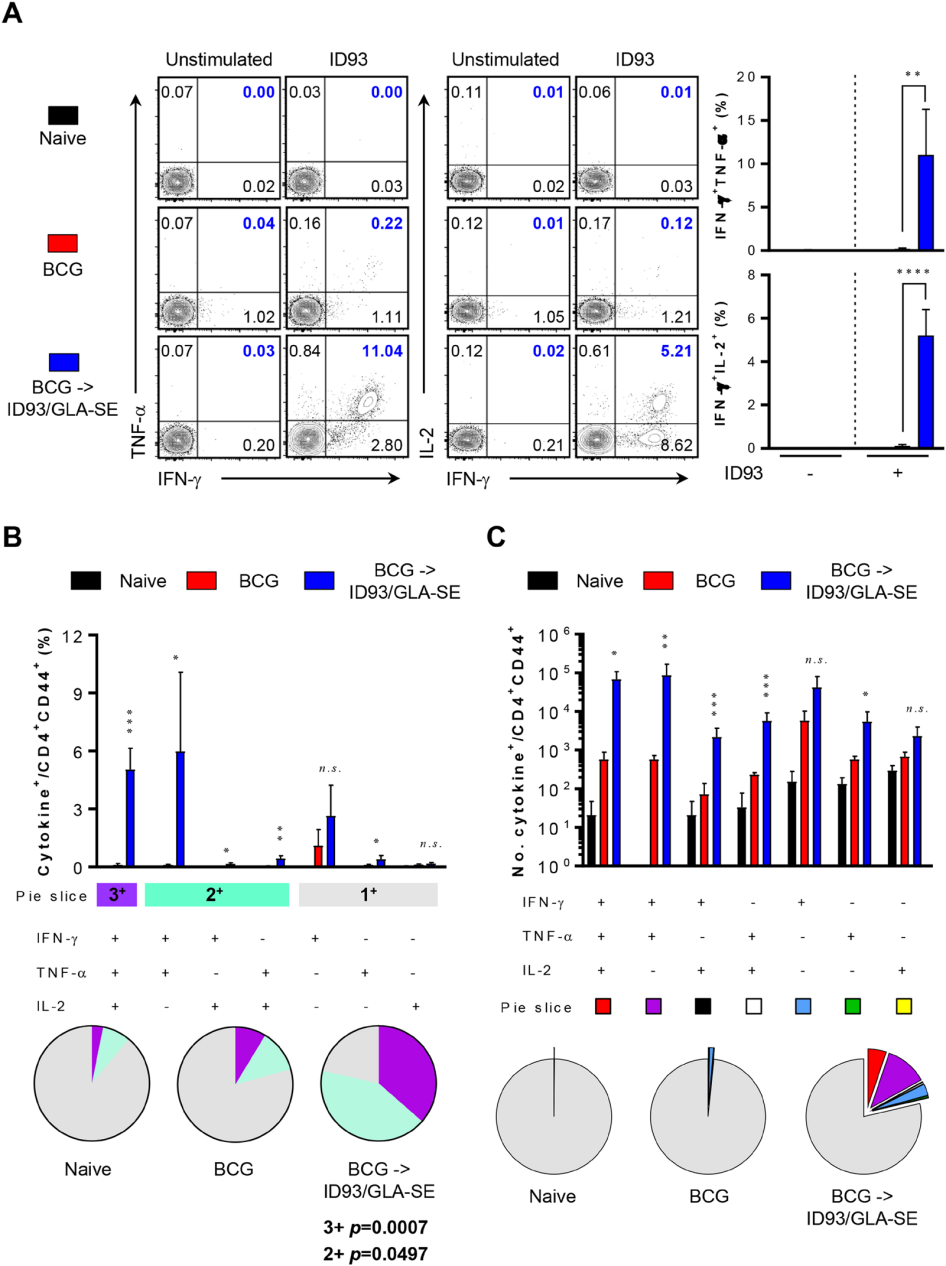
Quantitative and qualitative analysis of antigen-specific multifunctional CD4^+^ T-cells induced by ID93/GLA-SE immunisation in the lung. Four weeks after final immunisation, mice from each group (*n* = 4) were sacrificed, and lung cells were re-stimulated with or without ID93 (1 μg/ml) at 37 °C for 12 hours in the presence of GolgiStop and GolgiPlug. (**A**) The frequencies of ID93-specific IFN-γ^+^TNF-α^+^- or IFN-γ^+^IL-2^+^-producing CD4^+^CD44^+^ T-cells were determined by intracellular cytokine staining in the lungs of each immunised mice as representative dot plots with bar graphs. (**B**) ID93-stimulated lung cells from each immunised group were evaluated based on the percentage of total CD4^+^CD44^+^ T-cells with different patterns of cytokine production and described as bar graphs (upper). The pie charts summarise the fractions of triple (3^+^, purple), double (2^+^, light jade), and single (1^+^, grey) CD4^+^CD44^+^ T-cell producers of IFN-γ, TNF-α and IL-2 in each immunised group (lower). (**C**) The actual number of ID93-specific multifunctional T-cells among total CD4^+^CD44^+^ T-cells from each immunised group is represented as bar graphs (upper). The mean number of cytokine-positive cells is displayed as pie charts with multiple colour fractions, and the number of rest population in total CD4^+^CD44^+^ T-cells is described as grey fraction in the pie charts (lower). The experimental results are presented as the mean ± SD from 4 mice from each group. Statistically significant differences between the groups were determined using an unpaired Student’s *t* test. *n.s*.; not significant, *p < 0.05, **p < 0.01, ***p < 0.001, and ****p < 0.0001 comparing the BCG immunised mice and BCG-primed ID93/GLA-SE boosted mice.

**Figure 2:**
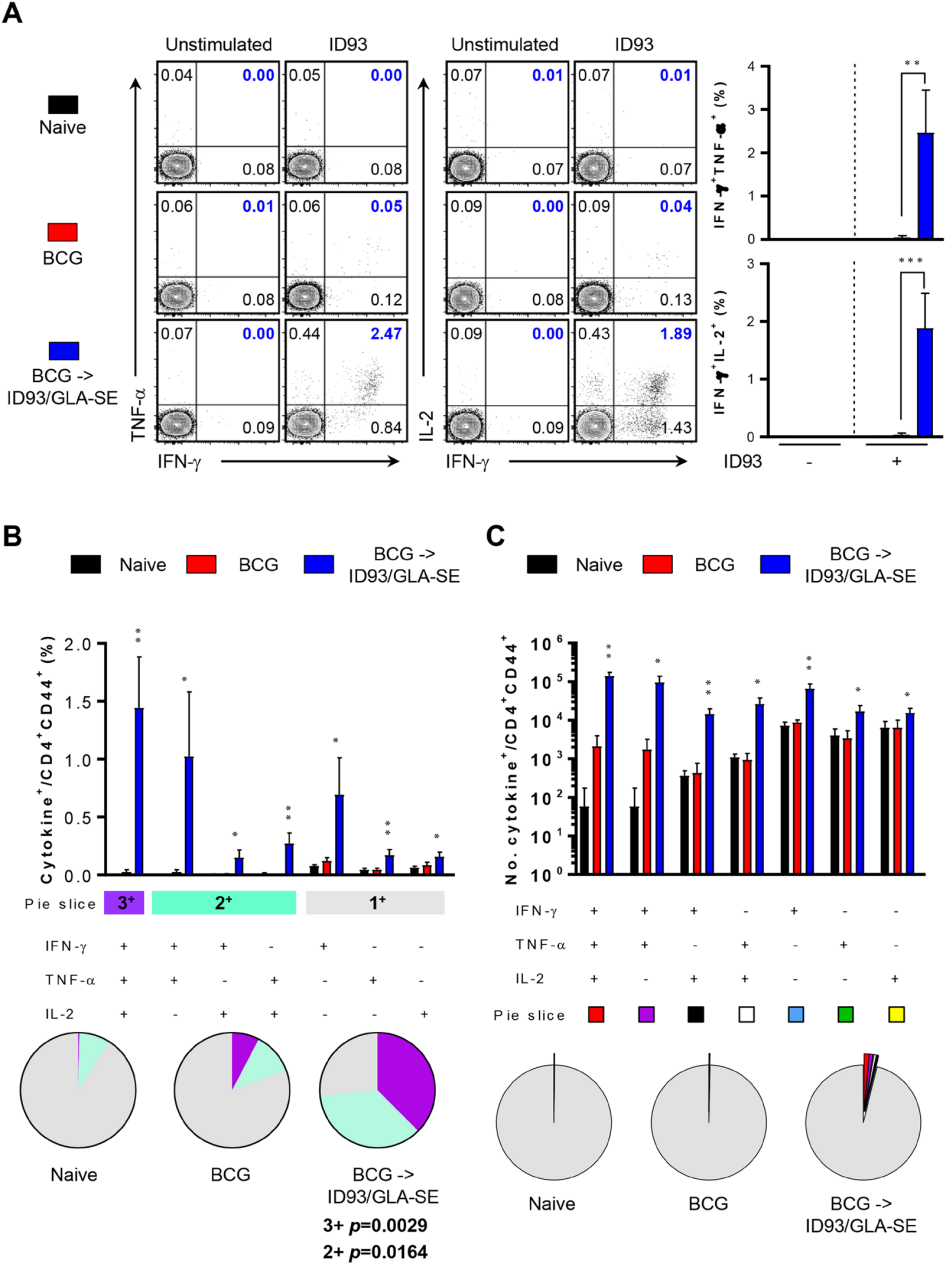
Induction of antigen-specific multifunctional T-cells based on quality and magnitude in spleen of ID93/GLA-SE-immunised mice. Similar to lung cells from each immunised mice (*n* = 4) depicted in Fig. 1, spleen cells were restimulated with ID93 (1 μg/ml) at 37 °C for 12 hours in the presence of GolgiStop and GolgiPlug. (**A**) The frequencies of ID93-specific IFN-γ^+^TNF-α^+^- or IFN-γ^+^IL-2^+^-producing CD4^+^CD44^+^ T-cells were determined by intracellular cytokine staining in the spleens of each immunised mice as representative dot plots with bar graphs. (**B**) ID93-stimulated spleen cells from each immunised group were evaluated based on the percentage of total CD4^+^CD44^+^ T-cells with different patterns of cytokine production and described as bar graphs (2B, upper). The pie charts summarise the fractions of triple (3^+^, purple), double (2^+^, light jade), and single (1^+^, grey) CD4^+^CD44^+^ T-cell producers of IFN-γ, TNF-α and IL-2 in each immunised group (2B, lower). (**C**) The actual number of ID93-specific multifunctional T-cells among total CD4^+^CD44^+^ T-cells from each immunised group was represented as the bar graphs (2C, upper). The mean number of cytokine-positive cells are displayed as pie charts with multiple colour fractions, and the number of rest population in total CD4^+^CD44^+^ T-cells was described as grey fraction in the pie charts (2 C, lower). The experimental results are presented as the mean ± SD from 4 mice from each group. Statistically significant differences between the groups were determined using an unpaired Student’s *t* test. *p < 0.05, **p < 0.01, and ***p < 0.001 comparing the BCG immunised mice and BCG-primed ID93/GLA-SE boosted mice.

We previously showed prophylactic efficacy of ID93/GLA-SE vaccine candidate, in the absence of BCG priming, where mice exhibited remarkable reduction of bacterial burden in the tested organs in addition to significantly attenuated lung inflammation following Mtb K infection^12^. In addition, *ex vivo* re-stimulation of cells from ID93/GLA-SE immunised mice with ID93 produced substantial IFN-γ in both lung and spleen in a dose-dependent manner^12^. In the present study, we show antigen-specific immune responses including cellular and humoral responses in BCG-vaccinated mice followed by immunisation with ID93/GLA-SE, where lung and spleen cells were re-stimulated with ID93 or PPD, and immune responses were compared with those of BCG vaccination alone control group (Figs 1 and 2, Supplementary Figs S2–S4).

After *ex vivo* stimulation of lung or spleen cells with ID93, cells from BCG-primed, ID93/GLA-SE boosted mice produced statistically significant polyfunctional T-cell responses including increased frequencies and absolute numbers of triple-positive (*p* < 0.001 for lung and *p* < 0.01 for spleen) and double-positive (*p* < 0.005 for lung and *p* < 0.05 for spleen) T-cells producing IFN-γ plus TNF-α and/or IL-2. In contrast, BCG immunised mice produced marginal T-cell responses in both organs (Figs 1 and 2). However, no differences in T-cell response between BCG-immunised group or in the BCG-primed, ID93/GLA-SE boosted group were detected in response to PPD *ex vivo* stimulation (Supplementary Figs S2 and S3), indicating that T-cell responses following ID93/GLA-SE immunisation were elicited in an ID93-specific manner prior to Mtb challenge. No increase of multifunctional CD8^+^ T-cell response was observed in response to either ID93 or PPD stimulation (data not shown).

Next, four weeks after the final immunisation antigen-specific antibody responses to PPD or ID93 were assessed in each group to determine whether immunisation elicited humoral responses. The ID93-specific IgG responses, including IgG1 and IgG2c, were significantly pronounced in the BCG-primed ID93/GLA-SE–boosted group compared to the BCG alone group (*p* < 0.0001) (Supplementary Fig. S4A). Interestingly, PPD-specific IgG2c in the sera from the BCG-primed ID93/GLA-SE–boosted group was detected (Supplementary Fig. S4B). Thus, these results show that ID93/GLA-SE immunisation in BCG-primed animals strongly elicit ID93-specific multifunctional CD4^+^ T-cell responses both in lung and spleen and elicit a potent antibody response, while BCG induced immune responses were limited in response to both antigens.

### Long-lasting protective efficacy following challenge with Mtb K in BCG-primed mice boosted with the ID93/GLA-SE vaccine candidate

**Figure 3:**
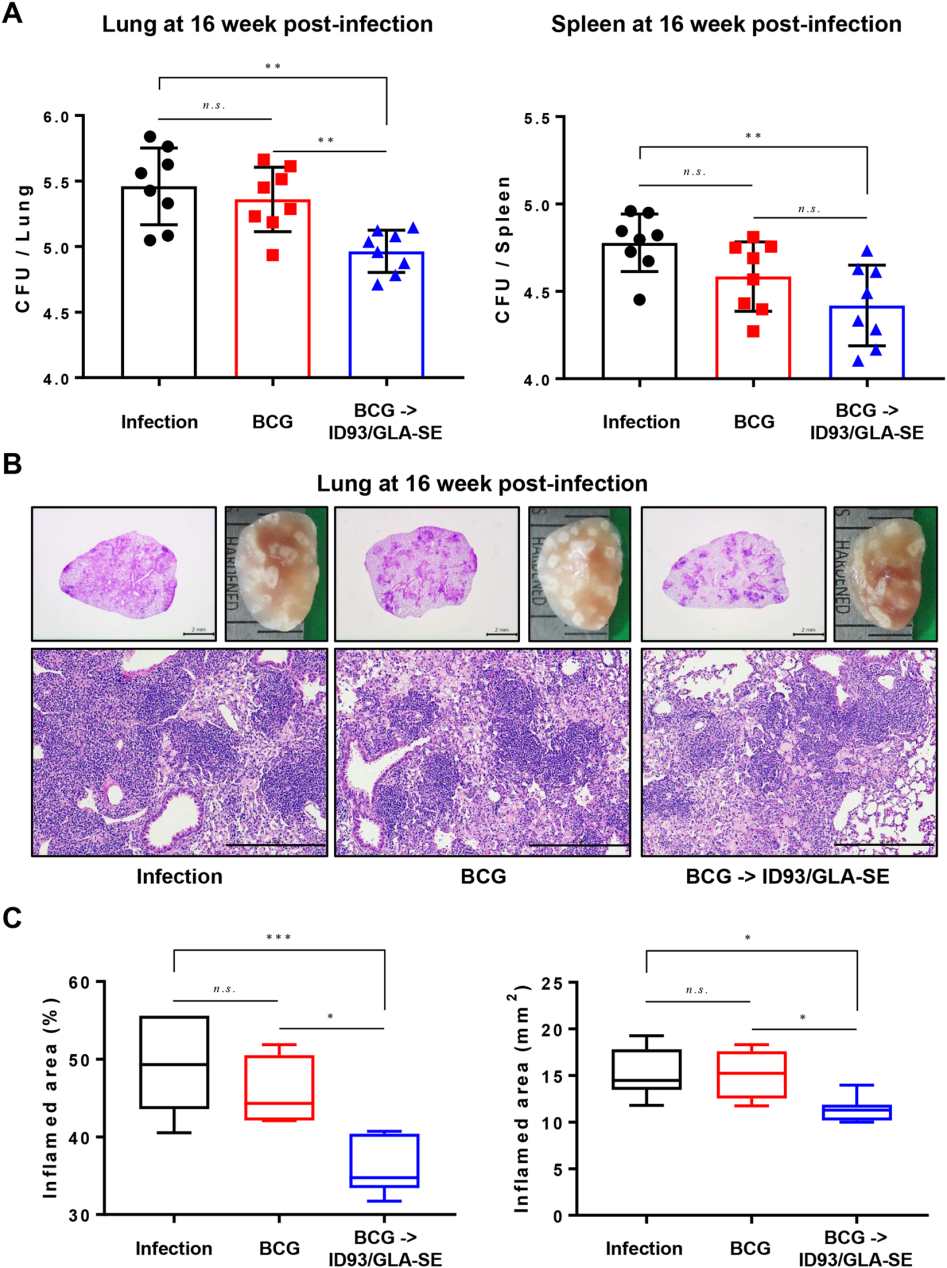
Long-term protective efficacy against the hypervirulent Mtb K strain using a prime/boost regimen with BCG and the ID93/GLA-SE vaccine candidate. (**A**) CFUs in the lungs and spleens of each group at 16 weeks post infection was analysed by enumerating the viable bacteria. (**B**) The superior lobes of the right lung of each immunised mouse were analysed using H&E staining (3B, upper left and bottom), and representative lung lobes were depicted as gross images (3B, upper right) at 16 weeks after Mtb K infection (1X: Scale bar = 2.0 mm, 10X: Scale bar = 0.5 mm). (**C**) The experimental results indicate the percentage of inflamed area and the size of lesions in the lung, and are described through box and whisker plots. Data from each immunised group (*n* = 8) at designated time point are described. One-way ANOVA followed by Tukey’s multiple comparison test was used to evaluate the significance. *n.s*.: not significant, *p < 0.05, **p < 0.01, and ***p < 0.001.

To compare the protective efficacy, in terms of reduction in bacterial load in mice, bacterial burdens of each group of mice were analysed at 16 weeks after challenge. Bacterial CFU in the lungs of BCG immunised mice showed no significant reduction against Mtb K strain challenge (Fig. 3A) at 16 weeks post-infection. However, ID93/GLA-SE significantly reduced the number of viable bacteria in the lungs compared to the infection control and to BCG only groups 16 weeks after challenge (*p* < 0.01 for both infection control and BCG control). The bacterial burden in spleen also showed similar pattern to CFU in lungs (Fig. 3A). Overall, the animals receiving ID93/GLA-SE immunisation following a BCG-prime showed long-term protective efficacy against K strain challenge in terms of bacterial reduction while BCG vaccination did not (Fig. 3A).

### Reduced lung inflammation following challenge with Mtb K in BCG-primed, ID93/GLA-SE boosted mice

One characteristic of Mtb K infection in C57BL/6 mice is the induction of exacerbated lung pathology 8 weeks after infection compared to Mtb H37Rv^25^. To evaluate protection against the extensive lung inflammation observed following infection with Mtb K, lungs were harvested from mice immunised with ID93/GLA-SE vaccination following priming with BCG, and lung pathology was assessed. To measure the extent of lung inflammation, histological preparations from each mouse were stained with H&E and the affected areas of inflammation relative to the total lung area were analysed.

Compared to the infection control and BCG-vaccination group, mice primed with BCG and boosted with ID93/GLA-SE had remarkably decreased granuloma-like lesions and reduced cellularity and size of the lungs when examined by gross pathology (Fig. 3B). Interestingly, the BCG-primed, ID93/GLA-SE boosted immunisation group showed significantly reduced inflammation compared to the infection control group (*p* < 0.001 for inflamed area percentage and *p* < 0.05 for inflamed area legions) and BCG-vaccination group (*p* < 0.05 for inflamed area percentage and *p* < 0.05 for inflamed area legions) in terms of inflammatory areas impacting the lung (Fig. 3C). Thus, ID93/GLA-SE immunisation conferred prevention of severe and extensive lung inflammation compared to that observed following BCG vaccination against Mtb K strain infection.

### Long-term maintenance of antigen-specific CD4^+^ multifunctional Th1 responses after Mtb challenge in BCG-primed, ID93/GLA-SE boosted mice

**Figure 4:**
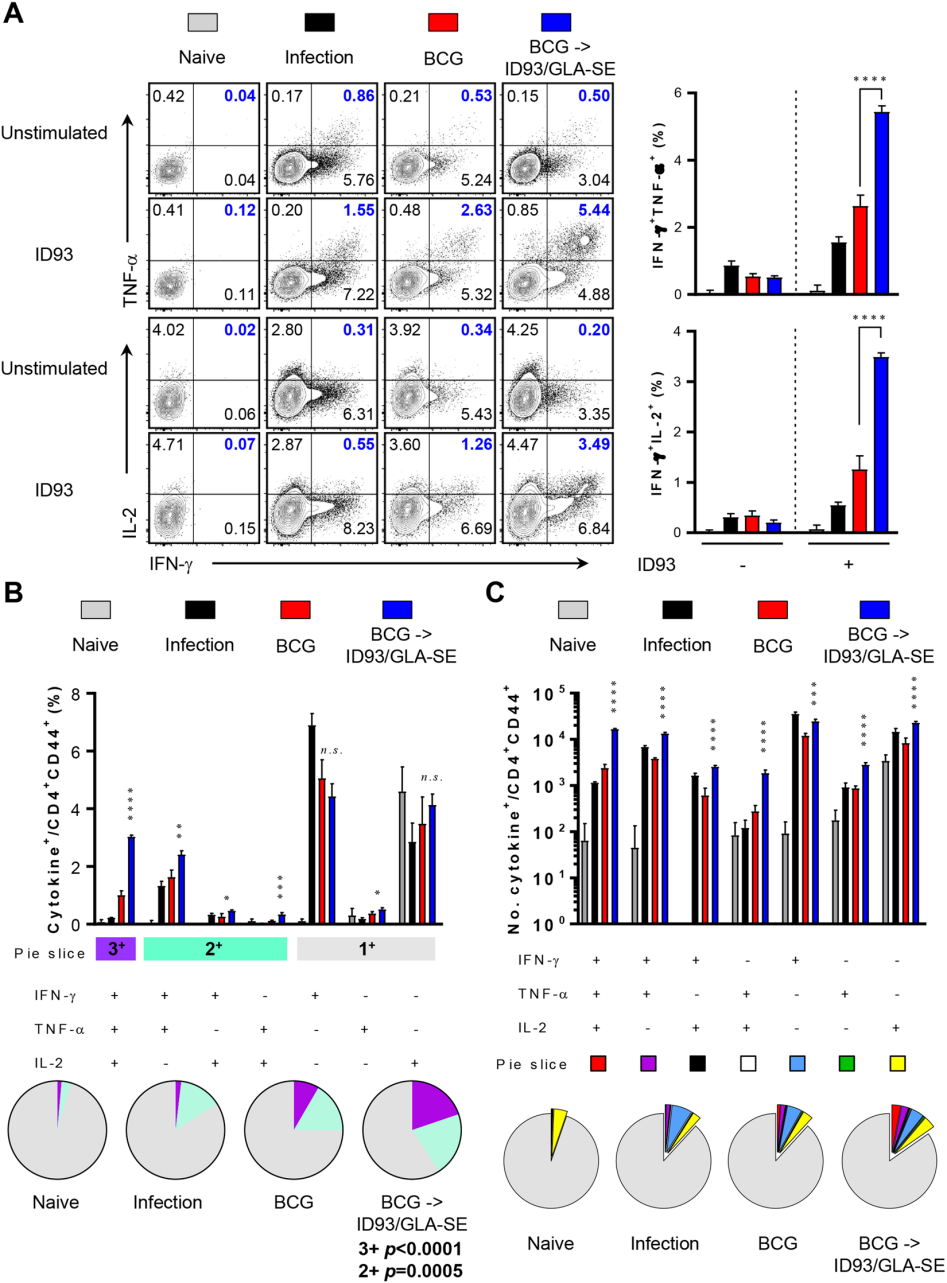
Long-lasting induction of antigen-specific polyfunctional CD4^+^ T-cell responses in the lungs of BCG primed, ID93/GLA-SE boosted mice following infection with the Mtb Beijing strain K. The mice in each group were sacrificed at 16 weeks post-infection, and lung cells were stimulated *ex vivo* with ID93 (1 μg/ml) at 37 °C for 12 hours in the presence of GolgiStop and GolgiPlug. (**A**) Upon stimulation with the ID93, the frequencies of Ag-specific CD4^+^CD44^+^ T-cells co-producing IFN-γ^+^TNF-α^+^ or IFN-γ^+^IL-2^+^ in the lung cells from each immunised group were evaluated using flow cytometry and described as representative dot plots and bar graphs. (**B**) ID93-stimulated lung cells from each immunised group were evaluated based on the percentage of total CD4^+^CD44^+^ T-cells with different patterns of cytokine production and described as bar graphs (4B, upper). The pie charts summarise the fractions of triple (3^+^, purple), double (2^+^, light jade), and single (1^+^, grey) CD4^+^CD44^+^ T-cell producers of IFN-γ, TNF-α and IL-2 in each immunised group (4B, lower). (**C**) The actual number of ID93-specific multifunctional T-cells among total CD4^+^CD44^+^ T-cells from each immunised group was represented as the bar graphs (4 C, upper). The mean number of cytokine-positive cells are displayed as pie charts with multiple colour fractions, and the number of rest population in total CD4^+^CD44^+^ T-cells was described as grey fraction in the pie charts (4C, lower). The experimental results are presented as the mean ± SD from 8 mice from each group. Statistically significant differences between the groups were determined using an unpaired Student’s *t* test. *n.s*.; not significant, *p < 0.05, **p < 0.01, ***p < 0.001, and ****p < 0.0001 comparing the BCG immunised mice and BCG-primed ID93/GLA-SE boosted mice.

**Figure 5:**
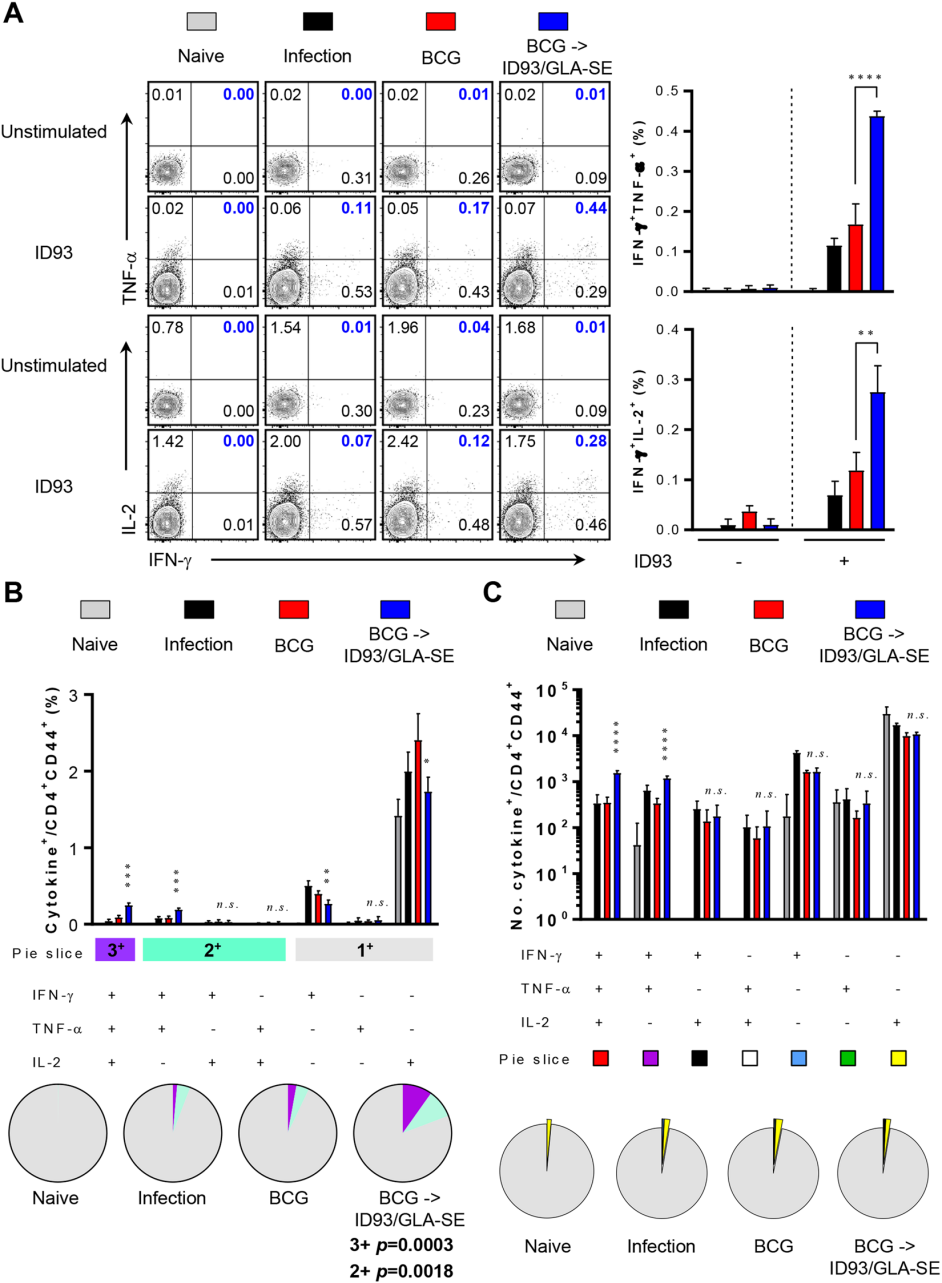
Long-lasting induction of antigen-specific polyfunctional CD4^+^ T-cell responses in the spleens of BCG primed, ID93/GLA-SE boosted mice following infection with the Mtb Beijing strain K. The prepared splenocytes were re-stimulated similarly to the lung cells. (**A**) Upon stimulation with the ID93, the frequencies of Ag-specific CD4^+^CD44^+^ T-cells co-producing IFN-γ^+^TNF-α^+^ or IFN-γ^+^IL-2^+^ in the spleen cells from each immunised group were evaluated using flow cytometry and described as representative dot plots and bar graphs. (**B**) ID93-stimulated spleen cells from each immunised group were evaluated based on the percentage of total CD4^+^CD44^+^ T-cells with different patterns of cytokine production and described as bar graphs (5B, upper). The pie charts summarise the fractions of triple (3^+^, purple), double (2^+^, light jade), and single (1^+^, grey) CD4^+^CD44^+^ T-cell producers of IFN-γ, TNF-α and IL-2 in each immunised group (5B, lower). (**C**) The actual number of ID93-specific multifunctional T-cells among total CD4^+^CD44^+^ T-cells from each immunised group was represented as the bar graphs (5C, upper). The mean number of cytokine-positive cells are displayed as pie charts with multiple colour fractions, and the number of rest population in total CD4^+^CD44^+^ T-cells was described as grey fraction in the pie charts (5C, lower). The experimental results are presented as the mean ± SD from 8 mice from each group. Statistically significant differences between the groups were determined using an unpaired Student’s *t* test. *n.s*.; not significant, *p < 0.05, **p < 0.01, ***p < 0.001, and ****p < 0.0001 comparing the BCG immunised mice and BCG-primed ID93/GLA-SE boosted mice.

We recently reported that durable protection of ID93/GLA-SE immunisation-induced anti-TB immunity was correlated with the sustained multifunctional CD4^+^ T-cell-mediated Th1 immune response after Mtb challenge^12,13^. To determine whether ID93-specific Th1 responses persisted in the context of the BCG prime, ID93/GLA-SE boosting strategy, we measured immune responses in the lungs of mice after Mtb K strain challenge (Figs 4 and 5). The frequency of ID93-specific triple positive (*p* < 0.0001 for lung and *p* < 0.001 for spleen) and double positive (*p* < 0.001 for lung and *p* < 0.01 for spleen) multifunctional CD4^+^ T-cells in the lung and spleen of mice in the BCG-primed, ID93/GLA-SE boosted group was maintained at 16 weeks after challenge with Mtb K (Figs 4 and 5). For PPD stimulation, BCG-primed immune responses seemed to be especially boosted in the lungs, not the spleens, by ID93/GLA-SE vaccination as evidenced by increased frequency of PPD-specific triple positive (*p* < 0.0001 for lung) and double positive (*p* < 0.001 for lung) multifunctional CD4^+^ T-cells (Supplementary Figs S5 and S6).

**Figure 6:**
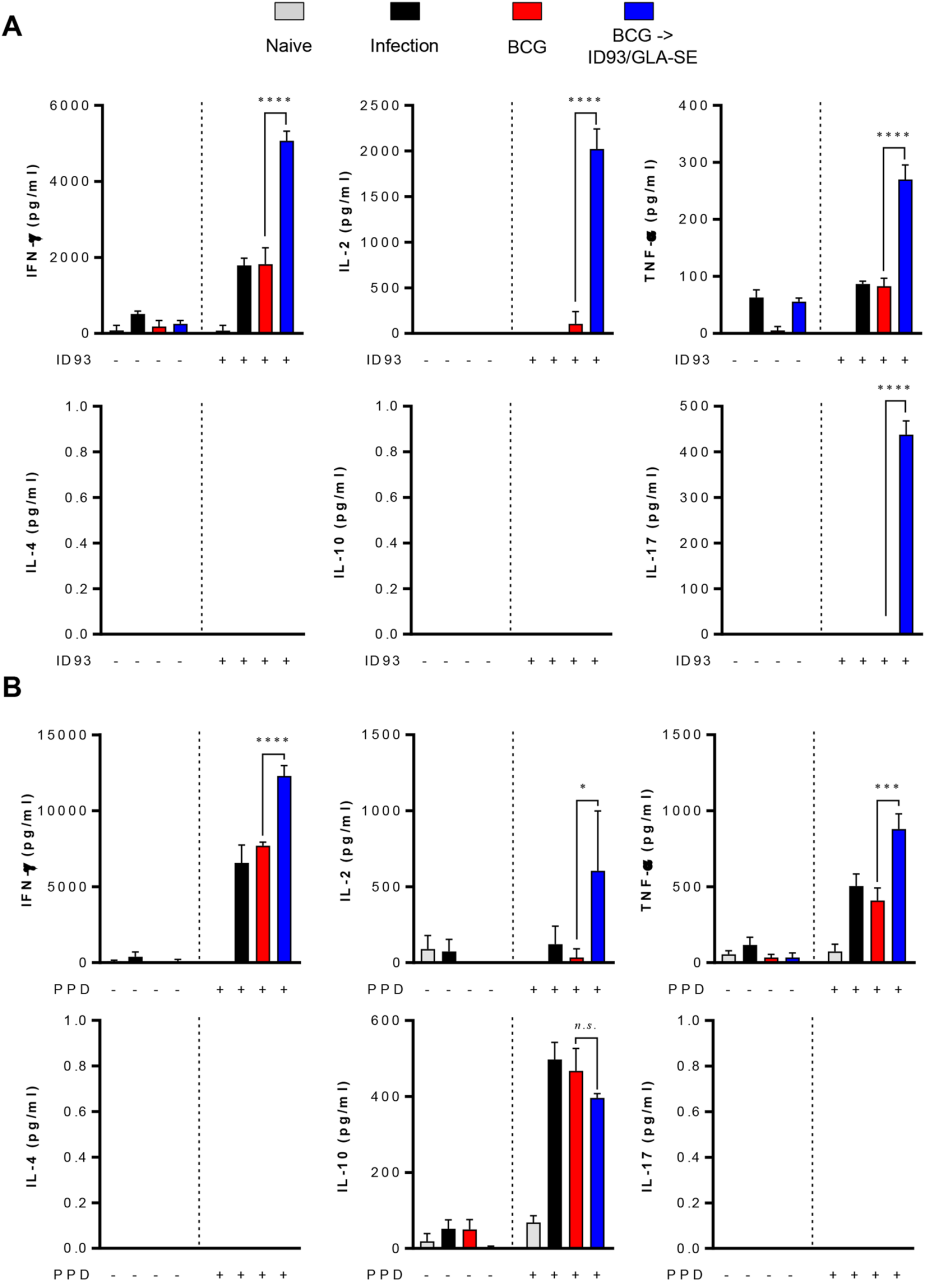
Protective cytokine profiles in the lungs of BCG primed, ID93/GLA-SE boosted mice 16 weeks following infection with the Mtb Beijing strain K. (**A**,**B**) Lung cells from naïve, infected, BCG-immunised and BCG-primed ID93/GLA-SE boosted mice (*n* = 8 mice/group at 16 weeks following infection) were stimulated with or without (**A**) ID93 (1 μg/ml) or (**B**) PPD (2 μg/ml) at 37 °C for 12 hours, and the supernatant was then collected and assayed for cytokines by ELISA. Statistically significant differences between the groups were determined using an unpaired Student’s *t* test. *n.s*.; not significant, *p < 0.05, ***p < 0.001, and ****p < 0.0001 comparing the BCG immunised mice and BCG-primed ID93/GLA-SE boosted mice.

Next, we investigated cytokine profiles in the lungs and spleens after re-stimulation with ID93 or PPD to determine whether Th1-mediated responses were maintained (Fig. 6 and Supplementary Fig. S7). Th1-associated cytokines including IFN-γ, TNF-α, and IL-2 but not Th2 cytokines, IL-4 and IL-10, were remarkably induced in the lungs from the BCG-primed ID93/GLA-SE boosted group in response to both ID93 (*p* < 0.0001 for all Th1-associated cytokines) and PPD *ex vivo* stimulation (*p* < 0.0001 for IFN-γ, *p* < 0.001 for TNF-α, and *p* < 0.05 for IL-2, respectively) (Fig. 6). Interestingly, IL-17A was induced only in the group that received the BCG-prime ID93/GLA-SE boost regimen in response to ID93 (Fig. 6A). No differences in IFN-γ or TNF-α production was detected between the BCG-vaccination group and BCG-prime ID93/GLA-SE boosted group in the spleen in response to either ID93 or PPD stimulation (Supplementary Figs S7A,B). Expression of IL-2, however was increased in response to ID93 in the BCG prime ID93/GLA-SE boosted group (*p* < 0.05) (Supplementary Fig. S7A).

## Discussion

In the present study, the long-term protective effect of the ID93/GLA-SE vaccine candidate was evaluated against the Beijing family strain, Mtb K, in a BCG prime-boost regimen. The protective immune response against the Mtb K strain, induced by this vaccine regimen, was characterised by a durable Th1-biased host response including antigen-specific CD4^+^ T-cells concomitantly producing IFN-γ, TNF-α, and IL-2 in both the lung and spleen (Figs 1 and 2) and potentially due to a Th1-biased ID93- and PPD-specific IgG2c humoral response (Supplementary Fig. S4). The effective boosting property of the ID93/GLA-SE vaccine within BCG-immunised mice was supported by a decrease in bacterial counts within the lung and spleen (Fig. 3A). Strikingly, excessive, irregular, and advanced granulomatous inflammation of lung from the infection control and BCG-vaccination groups were shown, whereas small, rounded and contained granulomas were found in the lung of mice vaccinated in mice received with BCG-priming boosting followed by ID93/GLA-SE immunisation (Fig. 3B). Although the ID93/GLA-SE vaccine candidate has been evaluated in many pre-clinical animal models, including a guinea pig model where ID93/GLA-SE was administered as a booster against the reference strain Mtb H37Rv^12,13,23^, this is the first study to evaluate the boosting effect of this vaccine candidate against a Beijing family strain of Mtb in BCG-immunised mice. The ID93/GLA-SE vaccine, once approved for use, would be particularly beneficial in East-Asian countries where this Beijing genotype is prevalent.

In our previous study, the ID93/GLA-SE vaccine induced protective efficacy when given as a prophylactic vaccine against Mtb K strain infection^12^, but the protection was less robust compared to the BCG vaccine. However, the improved potential of ID93/GLA-SE as a booster to the BCG vaccine in a prophylactic setting, not only reported in a guinea pig model^23^ but also shown in this study, suggests that the subunit vaccine is capable of re-stimulating the protective immunity upon BCG vaccination against infection with Mtb (Figs. 3, 4 and 5).

In fact, prior studies have shown that BCG vaccine efficacy is dependent on the infectious Mtb strain type^26,27^. The clinical isolate in South Korea, Mtb K, is a representative Beijing family strain from a TB outbreak in South Korean high schools^11^, where most of the students had previously received the BCG vaccine. The infectivity and pathogenicity of the virulent clinical Mtb K isolate has been studied in mice to characterise the differences compared to a standard Mtb H37Rv isolate^25^. In particular, W/Beijing Mtb strains exhibit an increased virulent phenotype displayed by faster growth, dampened protective Th1 cellular immune responses, and a higher relapse rate than the H37Rv strain *in vivo* due in part to an expansion of CD4^+^CD25^+^Foxp3^+^ regulatory T-cells compared to the H37Rv strain^25,27^. Moreover, BCG-efficacy against W/Beijing strains of Mtb wanes over time in animal models^27^ mimicking human responses to the vaccine.

Although the lack of reliable correlates of protection for TB makes a go/no-go decision for the advancement of TB vaccines difficult, the three multi-antigenic subunit vaccines, already in clinical development (M72/AS01_E_, H4:IC31, and ID93/GLA-SE), have commonly shown significant induction of Th1-polarised CD4^+^ T-cell responses characterised by the production of antigen-specific IFN-γ, TNF-α, and IL-2^28^. Furthermore, these vaccines induce protective efficacy in different animal models, measured by reduction in both bacterial CFU and lung inflammation^4,9,12,29^. The induction of antigen-specific CD4^+^ Th1 cellular responses after vaccination have therefore supported proof-of-concept for vaccine evaluation in Phase 1 clinical trials^10,30–34^.

In this study, significant induction of Th1-polarised Ag-specific multifunctional CD4^+^ T-cell responses and marked increase of Ag-specific humoral response were observed in BCG-vaccinated mice immunised with ID93/GLA-SE (Figs 1, 2 and supplementary Fig. S4). The immunisation conferred long-term protection against the virulent Mtb K, as evidenced by a significant CFU reduction in the lung and spleen, and by preventing excessive lung inflammation (Fig. 3).

It should be noted that long-lasting pulmonary control by prime-boost vaccination with BCG and ID93/GLA-SE against Mtb infection was accomplished (Fig. 3A). An enhanced level of pulmonary protection compared to that seen in the spleens was observed in the lungs of ID93/GLA-SE-boosted mice. First, the aerosol challenge of Mtb may lead to homing of antigen-specific T-cells to the lung engaging effector responses compared to the spleen. Secondly, while ID93-specific multifunctional CD4^+^ T-cells are induced in both the spleens and lungs of each immunised mouse prior to Mtb challenge (Figs 1 and 2), a larger number of ID93-responding multifunctional CD4^+^ T-cells appear to be maintained in lungs compared to that observed in the spleens after Mtb challenge (Figs 4 and 5). This suggests that the maintenance of ID93-specific multifunctional T-cell population, particularly in the lungs for at least 16 weeks post-infection may confer improved protection. Thirdly, a significantly higher magnitude of Th1-polarised cytokines such as IFN-γ, IL-2 and TNF-α, not Th2-associated cytokines, are induced following the *ex vivo* stimulation with both ID93 and PPD from cells in the lungs from ID93/GLA-SE-boosted mice (Fig. 6), but not in spleen except for IL-2 (Supplementary Fig. S7), which could contribute to better protection in the lung. Fourth, in addition to direct protective pulmonary contribution, boosting BCG with ID93/GLA-SE induced antigen-specific antibody responses (Supplementary Fig. S4). Unlike the guinea pigs data^23^, ID93-specific IgG1 and IgG2c responses were not found to be different in the BCG prime/boosted mouse study, representing an IgG2c/IgG1 mixed antibody response. Notably, PPD-specific IgG2c responses were significantly increased compared to IgG1 responses. This PPD-specific IgG2c biased response may be associated with an enhanced Th1-polarised response contributing to improved BCG primed/boosted protection against Mtb infection. Lastly, ID93-specific IL-17 production in the lungs was notably observed (Fig. 6A). Recently, the protective role of Th17 responses against Mtb has emerged in the literature by controlling Mtb growth and limiting extensive pathology in the lungs during Mtb infection^35–41^. In fact, IL-17-producing Th17 cells are considered to be particularly essential in vaccine-mediated immunity^35,42^ by rapidly recruiting CD4^+^IFN-γ^+^ T-cells to the site of infection, leading to earlier control of Mtb^35^. A previous study has shown that ID93/GLA-SE vaccination can elicit Th17 responses when given intranasally but not through intramuscular injection^43^. Although induced tissue-resident Th17 responses do not contribute to the protective efficacy in the murine subunit vaccination model^43^, ID93-specific IL-17 production may contribute to improved vaccine efficacy in animals that received ID93/GLA-SE as a boost in the BCG booster murine model which is employed for the first time (Figs 3 and 6). Nevertheless, the underlying mechanisms associated with improved protection conferred by BCG-primed ID93/GLA-SE boosting vaccination remains to be clearly defined.

Prior studies have shown that mice immunised with the ID93/GLA-SE candidate vaccine generate a Th1-biased response, with antigen-specific polyfunctional CD4^+^ T-cells. ID93/GLA-SE prophylactic immunisation has led to the protection of mice against challenge with either H37Rv^23,44^ or a multidrug-resistant TN5904 strain of Mtb^23^. Furthermore, boosting BCG with ID93/GLA-SE conferred long-term protection accompanied with ameliorated pathology against TB in guinea pig model^23^. Considering that promising results of heterologous prime/boost regimens such as BCG primed followed by boosting with adjuvanted antigens found in BCG and/or Mtb^45,46^, ID93 which is composed of antigens found in both Mtb and BCG could be capable of boosting BCG-primed immunity. Based on these results including our study, which strengthen the immunogenicity and protective efficacy of ID93/GLA-SE as a BCG booster, there is the potential for use of this vaccine candidate as a booster vaccine to improve the waning efficacy of the BCG vaccine. Since vaccination of BCG occurs within the first month of birth according to the BCG vaccination policy in South Korea, the efficacy testing of ID93/GLA-SE as a booster vaccine is ongoing in humans. Advances towards TB elimination by preventing Mtb transmission will require strenuous efforts including prevention of disease, where the impact of this BCG prime, ID93/GLA-SE boost vaccine regimen may be especially effective^23^.

Collectively, the capacity of T-cells to be expanded and differentiated into effector T-cells from central memory T-cells in response to BCG stimulation wanes with the increase of age in healthy children, suggesting that booster vaccines should be considered to maintain the antigen-specific T-cells and possibly enhance the duration of protection afforded by BCG^21^. ID93/GLA-SE was able to elicit robust and sustained Th1-biased immune responses including antigen-specific multifunctional CD4^+^ T-cell co-producing IFN-γ, TNF-α, and IL-2 as well as a high magnitude of antigen-specific Th1-biased antibodies. Our findings showed that ID93/GLA-SE vaccine as a BCG-prime booster confers a high level of long-term protection against the hypervirulent Mtb Beijing infection. These findings also showcase the potential utility of this vaccine candidate in East-Asian countries with the predominance of the Beijing genotype after BCG vaccination.

## Methods

### Ethics statement

All animal studies were carried out accordingly following the guidelines of the Korean Food and Drug Administration (KFDA). The experimental protocols used in this study were reviewed and approved by the Ethics Committee and Institutional Animal Care and Use Committee (Permit Number: 2015-0041) of the Laboratory Animal Research Center at Yonsei University College of Medicine (Seoul, Korea).

### Preparation of *Mycobacterium* spp

Mtb K strain was obtained from the strain collections at the Korean Institute of Tuberculosis (KIT, Osong, Chungchungbuk-do, Korea). *M. bovis* BCG (Pasteur strain 1173P2) was kindly provided by Dr. Brosch at the Pasteur Institute (Paris, France). These strains were cultured in Middlebrook 7H9 broth (Difco Laboratories, Detroit, MI) supplemented with 0.02% glycerol and 10% (vol/vol) oleic acid-albumin-dextrose-catalase (OADC, Becton Dickinson, Sparks, MD) for 28 days at 37 °C. Single cell suspensions of each strain were prepared as previously described^12^.

### Animals, immunisation, and challenge protocol

Specific pathogen-free 5-week-old female C57BL/6J mice were purchased from Japan SLC, Inc. (Shijuoka, Japan) and strictly maintained under barrier conditions in a BL-3 biohazard animal facility at the Yonsei University Medical Research Center. The animals were fed a sterile commercial mouse diet and provided with water *ad libitum*. The mice were monitored daily before and after immunisation and Mtb challenge, and none of the mice showed any clinical symptoms or illness during this experiment.

Immunisation with ID93 formulated in GLA-SE was performed as previously described^12^. Briefly, mice were immunised with BCG Pasteur 1173P2 via subcutaneous injection (2.0 × 10^5^ CFUs/mouse), then 10 weeks following the BCG immunisation, ID93/GLA-SE vaccine (1 μg ID93 antigen formulated in 5 μg of GLA-SE / injection) was given as a boost, intramuscularly, three times at 3-week intervals (Supplementary Fig. S1A). Four weeks after the final immunisation, the immunised mice were aerogenically challenged with the Mtb K strain as previously described^12^. Aerosol infection was performed using a Glas-Col aerosol apparatus (Terre Haute, IN, USA) adjusted to achieve an initial infectious dose of 200 CFUs. At 16 weeks post-challenge, mice from each group were euthanised to analyse bacterial load, histopathology, and immune responses including the frequency and number of multifunctional T-cells.

### Bacterial enumeration and histopathology

At 16 weeks following Mtb challenge, eight mice per group were euthanised with CO_2_, and lungs and spleens were homogenised. The number of viable bacteria was determined by plating serial dilutions of the organ homogenates onto Middlebrook 7H11 agar (Difco, USA) supplemented with 10% OADC (Difco, USA) and amphotericin B (Sigma Aldrich, USA). Colonies were counted after 4 weeks of incubation at 37 °C. For histopathological analysis, the middle cross-section from entire superior lobes of the right lung were stained with hematoxylin and eosin (H&E) and assessed for the severity of inflammation. The level of inflammation in the lungs was evaluated using by ImageJ (National Institutes of Health, USA) program, as previously described^47^. Briefly, for quantitative histopathological analysis of H&E staining, all slides were scanned using an Olympus BX43 microscope 4 × objective (Olympus Optical Co., Tokyo, Japan), and a pathologist assessed and quantified histopathology in a blinded manner. Each slide image per mouse was evaluated with the ToupTek Toup viewer program (Toup View Co., Zhejiang, China) to quantify the total size of the inflamed lesions in each mouse. The measured area is presented as mm^2^.

### Flow cytometry and intracellular cytokine staining

Single cell suspensions (1.5 × 10^6^ cells) from immunised or Mtb-infected mice were stimulated with PPD (2 μg/ml) or ID93 (1 μg/ml) at 37 °C for 2 hours. Then, stimulated-single cell suspensions were further incubated at 37 °C for 10 more hours in the presence of both GolgiPlug and GolgiStop (BD Biosciences). PPD was kindly provided by Dr. Michael Brennan at Aeras (Rockville, MD, USA). Cells were first washed with 2% FBS containing PBS and blocked with anti-CD16/32 (BD Biosciences) at 4 °C for 20 min. After the cells were stained with LIVE/DEAD^TM^ Fixable Viability Dye eFluor^TM^ 780 (ThermoFisher Scientific), and surface stained with Brilliant Violet (BV) 421-conjugated anti-CD4, peridinin chlorophyll (PerCP)-Cy5.5-conjugated anti-CD8, phycoerythrin (PE)-conjugated anti-CD44 (BD Biosciences) antibodies at 4 °C for 30 min and were washed. These cells were permeabilised and fixed with the Cytofix/Cytoperm kit (BD Biosciences) at 4 °C for 30 min. Then, cells were washed twice with Perm/Wash (BD Biosciences) and intracellularly stained with allophycocyanin (APC)-conjugated anti-IFN-γ, BV605-conjugated anti-TNF-α and PE-conjugated anti-IL-2 (Biolegend, San Diego, CA, USA) at 4 °C for 30 min. After washing three times with Perm/Wash, cells were fixed with IC Fixation buffer (eBioscience). Then, 2% FBS containing PBS-resuspended cells were analysed on FACSCanto II (BD Biosciences) using the commercially available software program FlowJo (Tree Star, Ashland, OR, USA). Next, the analysed antigen-specific multifunctional T-cells were either represented as percentages among total number of CD4^+^CD44^+^ T-cells or described as actual counts of cytokine-producing cells as bar graphs. Gating strategy for flow cytometry analysis is depicted in Supplementary Figure S1B. For pie charts analysis, the values summarise the fractions of triple (3^+^, purple), double (2^+^, light jade), and single (1^+^, grey) CD4^+^CD44^+^ T-cell producers of IFN-γ, TNF-α and IL-2 in each immunised group. The mean number of cytokine-positive cells is displayed as pie charts with multiple colour fractions, and the number of the rest of the population in total CD4^+^CD44^+^ T-cells was described as a grey fraction in the pie charts.

### Antibody titers in serum

96 well plates were coated with 1 μg/ml of PPD or ID93, and Ag-specific IgG, IgG1 and IgG2c responses in serum of immunised mice were measured as previously described^48^.

### Quantification of cytokines

Cytokines in *ex vivo*-isolated single cells from the lungs and spleens of infected mice were analysed by commercial ELISA kits according to the manufacturers’ instructions after stimulation with the PPD (2 μg/ml) or ID93 (1 μg/ml) for 24 hours at 37 °C. The ELISA was used for detecting IFN-γ, IL-2, IL-17, TNF-α, IL-4 and IL-10 in the culture supernatant. All ELISA kits were purchased from eBioscience, except for the IL-10 ELISA kit (BD Biosciences).

### Statistical analysis

Data for all experiments are presented as the mean ± standard deviation (SD). The significance of differences between samples was assessed by one-way ANOVA after Tukey’s post-test for multiple comparisons in more than three groups or by unpaired *t*-test in the selected two groups, followed by Shapiro-Wilk normality test using GraphPad Prism version 6.00 for Windows (GraphPad Software, La Jolla California USA, www.graphpad.com). Differences with each value of *p < 0.05, **p < 0.01, ***p < 0.001, or ****p < 0.0001 were considered statistically significant.

## Supplementary information

Supplementary Information

## References

[1] Global tuberculosis report 2018. Report No. ISBN 978-92-4-156564-6, (World Health Organization, Geneva, 2018).

[2] Nemes E (2018). Prevention of *M. tuberculosis* Infection with H4:IC31 Vaccine or BCG Revaccination. N Engl J Med.

[3] Van Der Meeren O (2018). Phase 2b Controlled Trial of M72/AS01E Vaccine to Prevent Tuberculosis. N Engl J Med.

[4] Reed SG (2009). Defined tuberculosis vaccine, Mtb72F/AS02A, evidence of protection in cynomolgus monkeys. Proc Natl Acad Sci USA.

[5] Tsenova L (2006). Evaluation of the Mtb72F polyprotein vaccine in a rabbit model of tuberculous meningitis. Infect Immun.

[6] Irwin SM (2005). Tracking antigen-specific CD8 T lymphocytes in the lungs of mice vaccinated with the Mtb72F polyprotein. Infect Immun.

[7] Hoang T (2013). ESAT-6 (EsxA) and TB10.4 (EsxH) based vaccines for pre- and post-exposure tuberculosis vaccination. PLoS One.

[8] Skeiky YA (2010). Non-clinical efficacy and safety of HyVac4:IC31 vaccine administered in a BCG prime-boost regimen. Vaccine.

[9] Billeskov R, Elvang TT, Andersen PL, Dietrich J (2012). The HyVac4 subunit vaccine efficiently boosts BCG-primed anti-mycobacterial protective immunity. PLoS One.

[10] Penn-Nicholson A (2018). Safety and immunogenicity of the novel tuberculosis vaccine ID93 + GLA-SE in BCG-vaccinated healthy adults in South Africa: a randomised, double-blind, placebo-controlled phase 1 trial. Lancet Respir Med.

[11] Kim SJ (2001). Transmission of *Mycobacterium tuberculosis* among high school students in Korea. Int J Tuberc Lung Dis.

[12] Cha SB (2016). Pulmonary immunity and durable protection induced by the ID93/GLA-SE vaccine candidate against the hyper-virulent Korean Beijing *Mycobacterium tuberculosis* strain K. Vaccine.

[13] Baldwin SL (2016). Protection and Long-Lived Immunity Induced by the ID93/GLA-SE Vaccine Candidate against a Clinical *Mycobacterium tuberculosis* Isolate. Clin Vaccine Immunol.

[14] Luo T (2015). Southern East Asian origin and coexpansion of *Mycobacterium tuberculosis* Beijing family with Han Chinese. Proc Natl Acad Sci USA.

[15] Merker M (2015). Evolutionary history and global spread of the *Mycobacterium tuberculosis* Beijing lineage. Nat Genet.

[16] Parwati I, van Crevel R, van Soolingen D (2010). Possible underlying mechanisms for successful emergence of the *Mycobacterium tuberculosis* Beijing genotype strains. Lancet Infect Dis.

[17] Brewer TF (2000). Preventing tuberculosis with bacillus Calmette-Guerin vaccine: a meta-analysis of the literature. Clin Infect Dis.

[18] Colditz GA (1994). Efficacy of BCG vaccine in the prevention of tuberculosis. Meta-analysis of the published literature. Jama.

[19] Mangtani P (2014). Protection by BCG vaccine against tuberculosis: a systematic review of randomized controlled trials. Clin Infect Dis.

[20] Roy A (2014). Effect of BCG vaccination against *Mycobacterium tuberculosis* infection in children: systematic review and meta-analysis. Bmj.

[21] Whittaker E, Nicol MP, Zar HJ, Tena-Coki NG, Kampmann B (2018). Age-related waning of immune responses to BCG in healthy children supports the need for a booster dose of BCG in TB endemic countries. Sci Rep.

[22] Larsen, S. E. *et al*. Enhanced Anti-*Mycobacterium tuberculosis* Immunity over Time with Combined Drug and Immunotherapy Treatment. *Vaccines (Basel)* **6** (2018). 10.3390/vaccines6020030 PMC6027321 29795025

[23] Bertholet S (2010). A defined tuberculosis vaccine candidate boosts BCG and protects against multidrug-resistant *Mycobacterium tuberculosis*. Sci Transl Med.

[24] Coler RN (2013). Therapeutic immunization against *Mycobacterium tuberculosis* is an effective adjunct to antibiotic treatment. J Infect Dis.

[25] Kim WS (2015). Virulence-Dependent Alterations in the Kinetics of Immune Cells during Pulmonary Infection by *Mycobacterium tuberculosis*. PLoS One.

[26] Jeon BY (2008). *Mycobacterium bovis* BCG immunization induces protective immunity against nine different *Mycobacterium tuberculosis* strains in mice. Infect Immun.

[27] Ordway DJ (2011). *Mycobacterium bovis* BCG-mediated protection against W-Beijing strains of *Mycobacterium tuberculosis* is diminished concomitant with the emergence of regulatory T cells. Clin Vaccine Immunol.

[28] Rodo MJ (2019). A comparison of antigen-specific T cell responses induced by six novel tuberculosis vaccine candidates. PLoS Pathog.

[29] Skeiky YA (2004). Differential immune responses and protective efficacy induced by components of a tuberculosis polyprotein vaccine, Mtb72F, delivered as naked DNA or recombinant protein. J Immunol.

[30] Leroux-Roels I (2013). Improved CD4(+) T cell responses to *Mycobacterium tuberculosis* in PPD-negative adults by M72/AS01 as compared to the M72/AS02 and Mtb72F/AS02 tuberculosis candidate vaccine formulations: a randomized trial. Vaccine.

[31] Norrby M (2017). Safety and immunogenicity of the novel H4:IC31 tuberculosis vaccine candidate in BCG-vaccinated adults: Two phase I dose escalation trials. Vaccine.

[32] Geldenhuys H (2015). The tuberculosis vaccine H4:IC31 is safe and induces a persistent polyfunctional CD4 T cell response in South African adults: A randomized controlled trial. Vaccine.

[33] Coler RN (2018). The TLR-4 agonist adjuvant, GLA-SE, improves magnitude and quality of immune responses elicited by the ID93 tuberculosis vaccine: first-in-human trial. NPJ Vaccines.

[34] Derrick SC, Yabe IM, Yang A, Morris SL (2011). Vaccine-induced anti-tuberculosis protective immunity in mice correlates with the magnitude and quality of multifunctional CD4 T cells. Vaccine.

[35] Khader SA (2007). IL-23 and IL-17 in the establishment of protective pulmonary CD4+ T cell responses after vaccination and during *Mycobacterium tuberculosis* challenge. Nat Immunol.

[36] Wozniak TM, Saunders BM, Ryan AA, Britton WJ (2010). *Mycobacterium bovis* BCG-specific Th17 cells confer partial protection against *Mycobacterium tuberculosis* infection in the absence of gamma interferon. Infect Immun.

[37] Scriba TJ (2008). Distinct, specific IL-17- and IL-22-producing CD4+ T cell subsets contribute to the human anti-mycobacterial immune response. J Immunol.

[38] Okamoto Yoshida Y (2010). Essential role of IL-17A in the formation of a mycobacterial infection-induced granuloma in the lung. J Immunol.

[39] Khader SA (2011). IL-23 is required for long-term control of *Mycobacterium tuberculosis* and B cell follicle formation in the infected lung. J Immunol.

[40] Freches D (2013). Mice genetically inactivated in interleukin-17A receptor are defective in long-term control of *Mycobacterium tuberculosis* infection. Immunology.

[41] Gopal R (2014). Unexpected role for IL-17 in protective immunity against hypervirulent *Mycobacterium tuberculosis* HN878 infection. PLoS Pathog.

[42] Desel C (2011). Recombinant BCG DeltaureC hly+ induces superior protection over parental BCG by stimulating a balanced combination of type 1 and type 17 cytokine responses. J Infect Dis.

[43] Orr MT (2015). Mucosal delivery switches the response to an adjuvanted tuberculosis vaccine from systemic TH1 to tissue-resident TH17 responses without impacting the protective efficacy. Vaccine.

[44] Baldwin SL (2012). The importance of adjuvant formulation in the development of a tuberculosis vaccine. J Immunol.

[45] Dietrich J, Billeskov R, Doherty TM, Andersen P (2007). Synergistic effect of bacillus calmette guerin and a tuberculosis subunit vaccine in cationic liposomes: increased immunogenicity and protection. J Immunol.

[46] Rouanet C, Debrie AS, Lecher S, Locht C (2009). Subcutaneous boosting with heparin binding haemagglutinin increases BCG-induced protection against tuberculosis. Microbes Infect.

[47] Kwon KW (2017). Novel vaccine potential of Rv3131, a DosR regulon-encoded putative nitroreductase, against hyper-virulent *Mycobacterium tuberculosis* strain K. Sci Rep.

[48] Kwon KW (2019). Vaccine efficacy of a *Mycobacterium tuberculosis* Beijing-specific proline-glutamic acid (PE) antigen against highly virulent outbreak isolates. Faseb j.

