# Supplementary material for: Long-term protective efficacy with a BCG-prime ID93/GLA-SE boost regimen against the hyper-virulent Mycobacterium tuberculosis strain K in a mouse model
Source: Sci Rep. 2019 Oct 29;9:15560. doi: 10.1038/s41598-019-52146-0 (PMC6820558; doi:10.1038/s41598-019-52146-0)
Supplement: Supplementary file 1 — Supplementary Information [file 41598_2019_52146_MOESM1_ESM.pdf]

Long-term protective efficacy with a BCG-prime ID93/GLA-SE boost regimen against the hyper-virulent *Mycobacterium tuberculosis* strain K in a mouse model

Kee Woong Kwon[a], Ara Lee[b], Sasha E. Larsen[c], Susan L. Baldwin[c], Rhea N. Coler[c,d,e], Steven G. Reed[c], Sang-Nae Cho[a], Sang-Jun Ha[b], Sung Jae Shin[a,*]

[a]Department of Microbiology, Institute for Immunology and Immunological Disease, Brain Korea 21 PLUS Project for Medical Science, Yonsei University College of Medicine, Seoul 03722, South Korea, [b]Department of Biochemistry, College of Life Science & Biotechnology, Yonsei University, Seoul 03722, South Korea, [c]Infectious Disease Research Institute, 1616 Eastlake Ave E, Suite 400, Seattle, WA 98102, USA, [d]Department of Global Health, University of Washington, Seattle, USA, [e]PAI Life Sciences Inc., Seattle, USA

* Correspondence to Sung Jae Shin, Department of Microbiology, Yonsei University College of Medicine, 50-1 Yonsei-ro, Seodaemun-gu, Seoul 03722, Korea, Tel: +82-2-2228-1813, Fax: +82-2-392-9310, e-mail: sjshin@yuhs.ac

Supplementary Information

Supplementary figures

Figure S1. Experimental design and gating strategy for flow cytometry analysis.

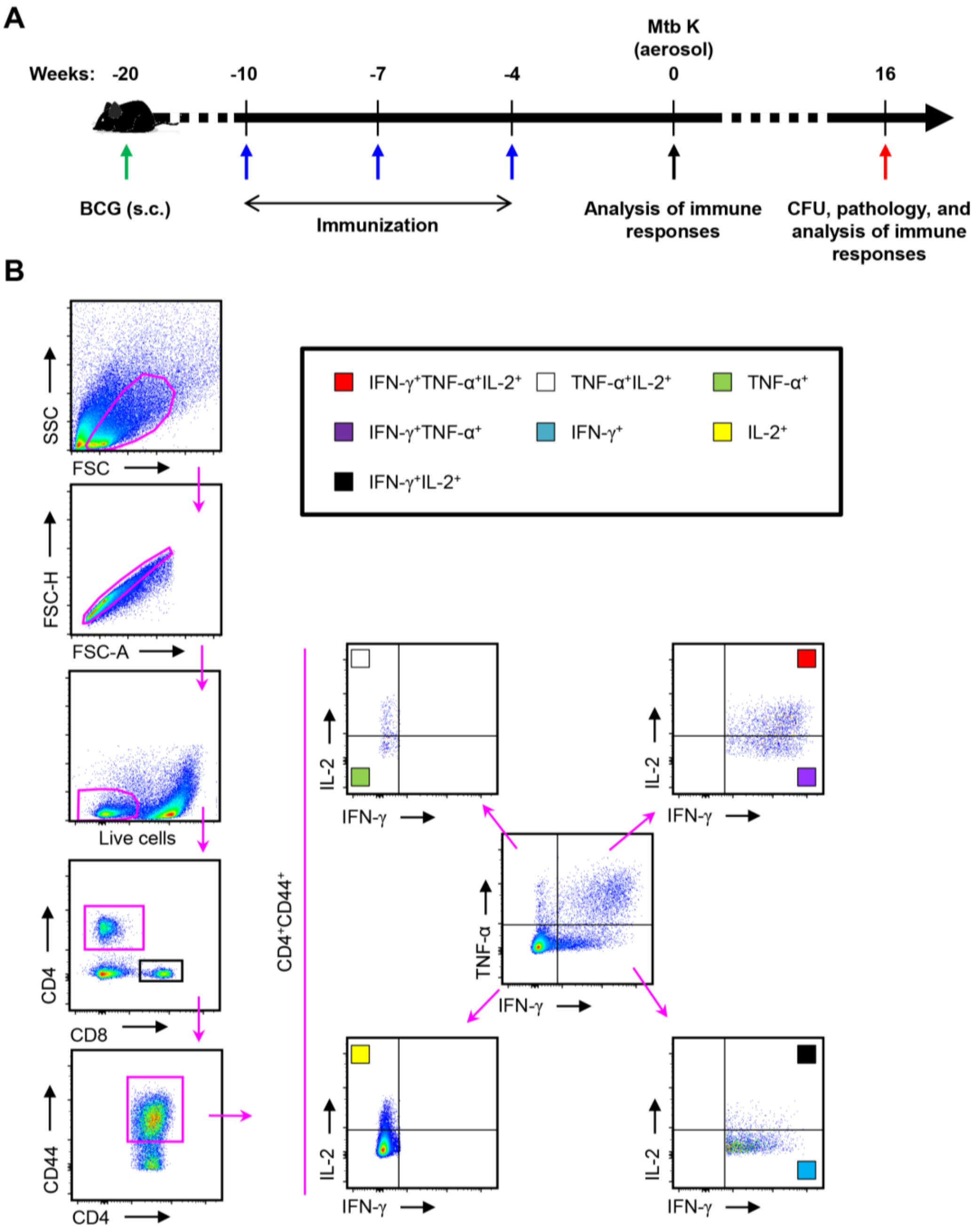

(A) Scheme of the experimental design for ID93/GLA-SE vaccine testing. Mice (*n* = 12/group)

were immunised with BCG subcutaneous injection 10 weeks before the first subunit vaccination (green arrow). After 10 weeks, mice were boosted with ID93/GLA-SE intramuscular injections three times at three weeks intervals (blue arrows). Prior to Mtb K infection, immune cells of lung and spleen from each immunised group were analysed (black arrow). After Mtb K infection, immunological analysis was performed, and bacterial counts and histopathological analysis in each immunised group were evaluated at the indicated time point (red arrow). (B) The mice in each group were sacrificed, and their lung and spleen cells were re-stimulated with ID93 (1 μg/ml) or PPD (2 μg/ml) at 37°C for 12 hours in the presence of GolgiStop and GolgiPlug. The gating strategy used to identify Ag-specific multifunctional T-cell populations.

Figure S2. Induction of PPD-specific polyfunctional CD4^{+} T-cells based on quality and magnitude in the lungs of the BCG-primed, ID93/GLA-SE-boosted mice.

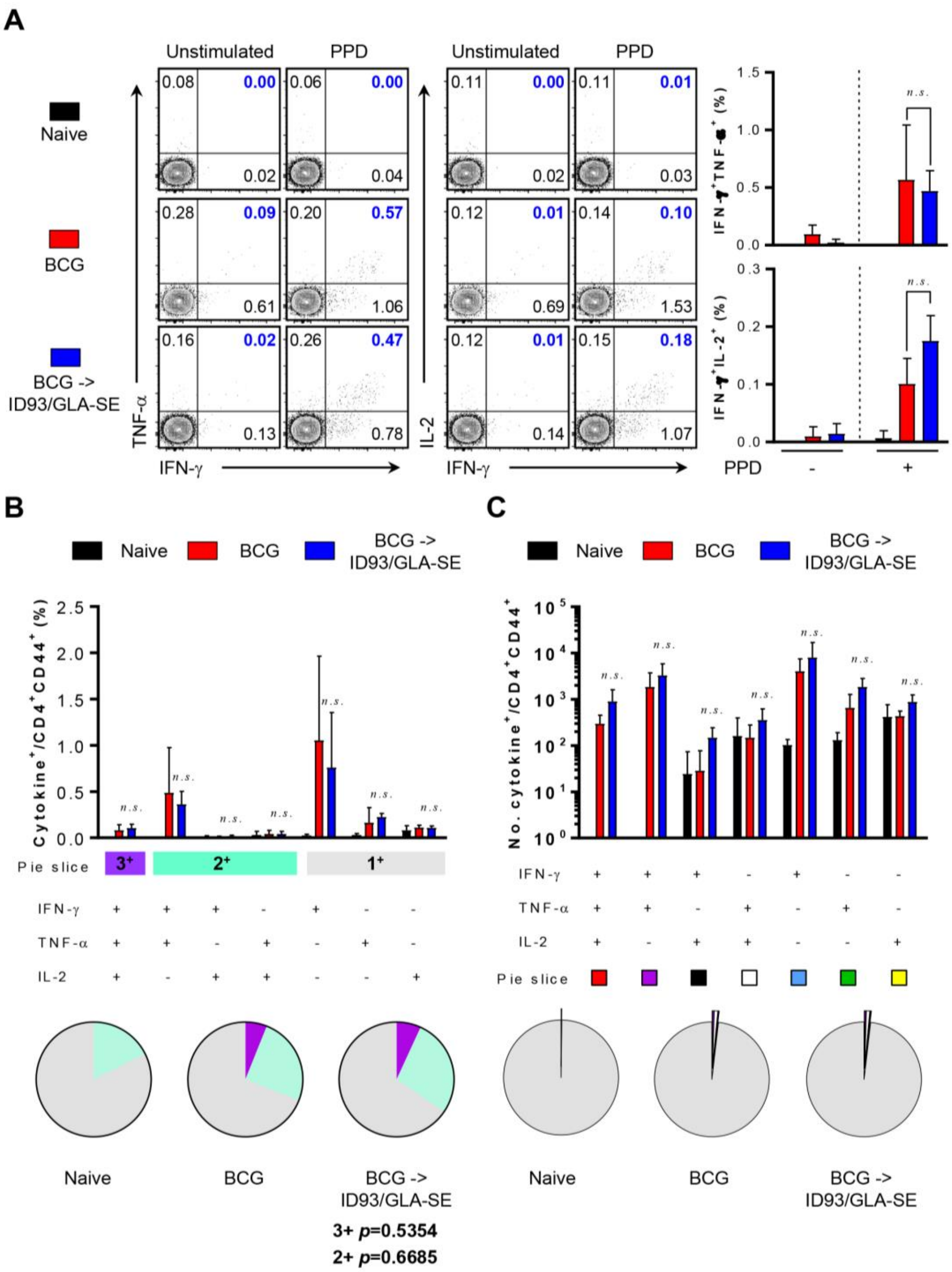

Four weeks after final immunisation, mice from each group (n = 4) were sacrificed, and lung cells were prepared as described in the materials and methods section. (A) The frequencies of

PPD-specific IFN-γ^+TNF-α^+- or IFN-γ^+IL-2^+-producing $CD4^+CD44^+$ T-cells were determined by intracellular cytokine staining in the lungs of each immunised mice as representative dot plots and bar graphs. (B) PPD-stimulated lung cells from each immunised group were evaluated based on the percentage of total $CD4^+CD44^+$ T-cells with different patterns of cytokine production and described as bar graphs (S2B, upper). The pie charts summarise the fractions of triple (3^+, purple), double (2^+, light jade), and single (1^+, grey) $CD4^+CD44^+$ T-cell producers of IFN-γ, TNF-α and IL-2 in each immunised group (S2B, lower). (C) The actual number of PPD-specific polyfunctional $CD4^+$ T-cells among total $CD4^+CD44^+$ T-cells from each immunised group was represented as the bar graphs (S2C, upper). The mean number of cytokine-positive cells are displayed as pie charts with multiple colour fractions, and the number of rest population in total $CD4^+CD44^+$ T-cells was described as grey fraction in the pie charts (S2C, lower). The experimental results are presented as the mean ± SD from 4 mice from each group. Statistically significant differences between the groups were determined using an unpaired Student's *t* test. *n.s.*; not significant comparing the BCG immunised mice and BCG-primed ID93/GLA-SE boosted mice.

Figure S3. Induction of PPD-specific polyfunctional CD4^{+} T-cells based on quality and magnitude in the spleen of the BCG primed, ID93/GLA-SE-boosted group.

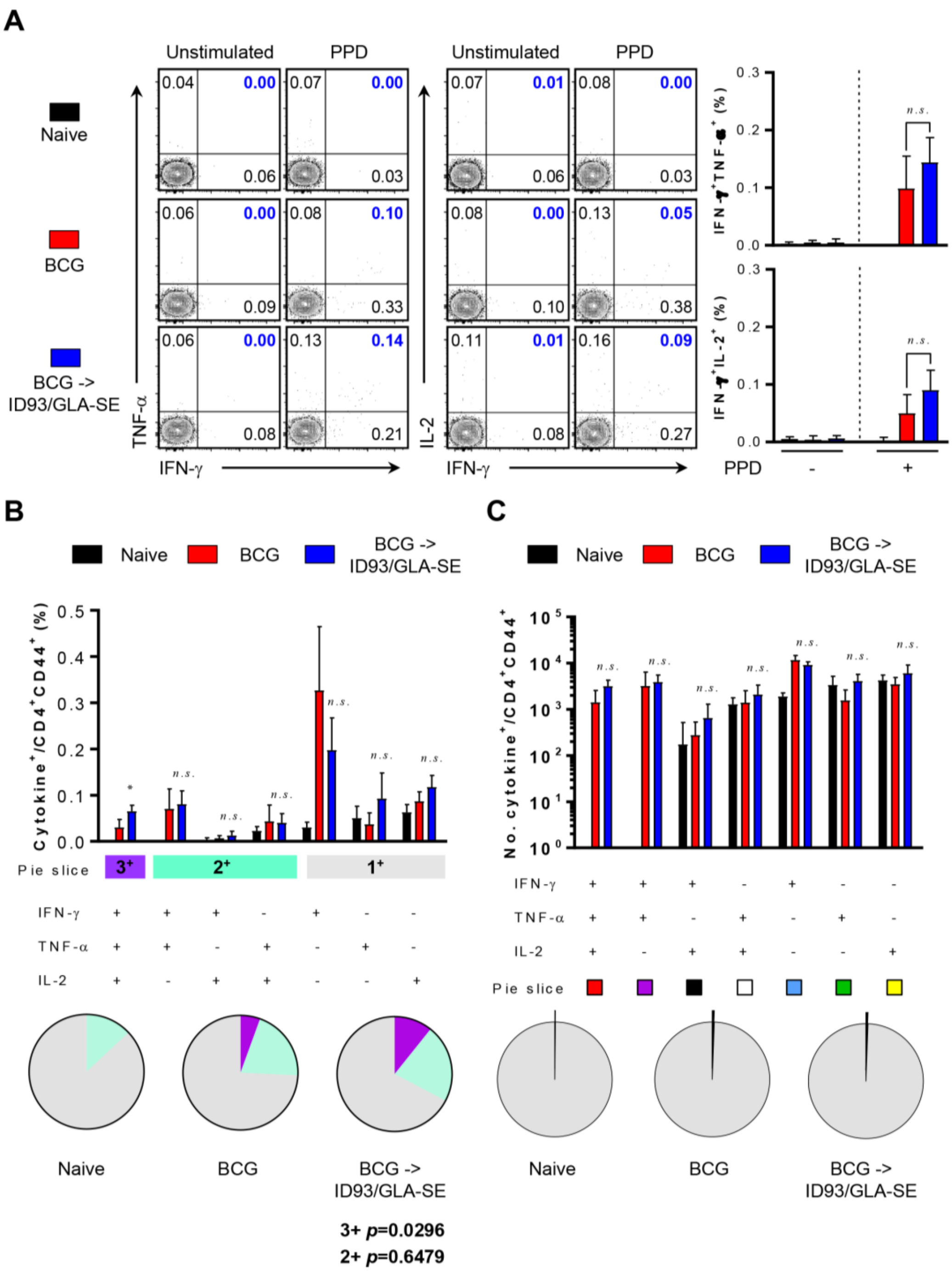

(A) The frequencies of PPD-specific IFN-γ^{+}TNF-α^{+}- or IFN-γ^{+}IL-2^{+}-producing CD4^{+}CD44^{+} T-cells were determined by intracellular cytokine staining in the spleens of immunised mice as

representative dot plots and bar graphs. (B) PPD-stimulated spleen cells from each immunised group were evaluated based on the percentage of total $CD4^+CD44^+$ T-cells with different patterns of cytokine production and described as bar graphs (S3B, upper). The pie charts summarise the fractions of triple (3^+, purple), double (2^+, light jade), and single (1^+, grey) $CD4^+CD44^+$ T-cell producers of IFN-γ, TNF-α and IL-2 in each immunised group (S3B, lower). (C) The actual number of PPD-specific polyfunctional T-cells among total $CD4^+CD44^+$ T-cells from each immunised group was represented as the bar graphs (S3C, upper). The mean number of cytokine-positive cells is displayed as pie charts with multiple colour fractions, and the number of rest population in total $CD4^+CD44^+$ T-cells was described as the grey fraction in the pie charts (S3C, lower). The experimental results are presented as the mean ± SD from 4 mice from each group. Statistically significant differences between the groups were determined using an unpaired Student's *t* test. *n.s.*; not significant, * $p<0.05$ comparing the BCG immunised mice and BCG-primed ID93/GLA-SE boosted mice.

Figure S4. Antigen-specific IgG responses in each immunised group.

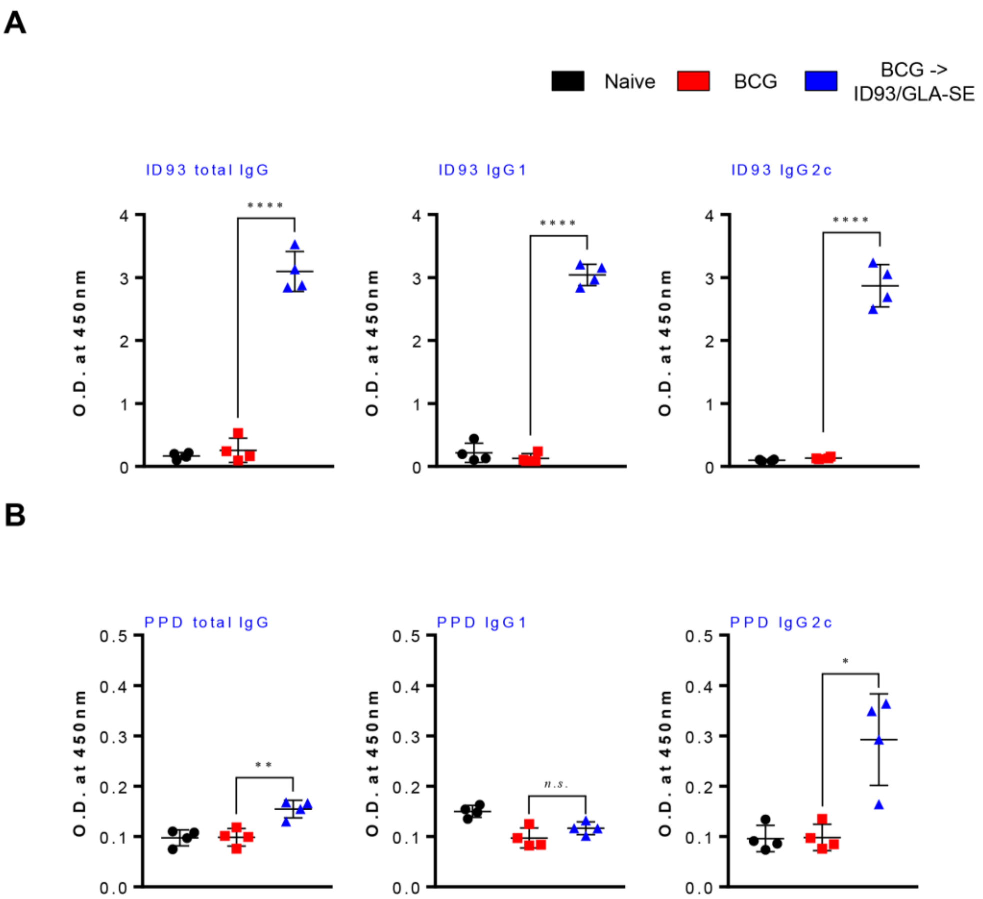

Antigen-specific antibody responses against ID93 (A) or PPD (B) in each group at 4 weeks after the last immunisation, as determined in serum samples. Statistically significant differences between the groups were determined using an unpaired Student's *t* test. *n.s.*; not significant, * $p<0.05$, ** $p<0.01$, and **** $p<0.0001$ comparing the BCG immunised mice and BCG-primed ID93/GLA-SE boosted mice.

Figure S5. Improved immune responses in the lungs of BCG-primed, ID93/GLA-SE boosted mice, represented by PPD-specific polyfunctional CD4+ T-cells after infection with Mtb Beijing strain K.

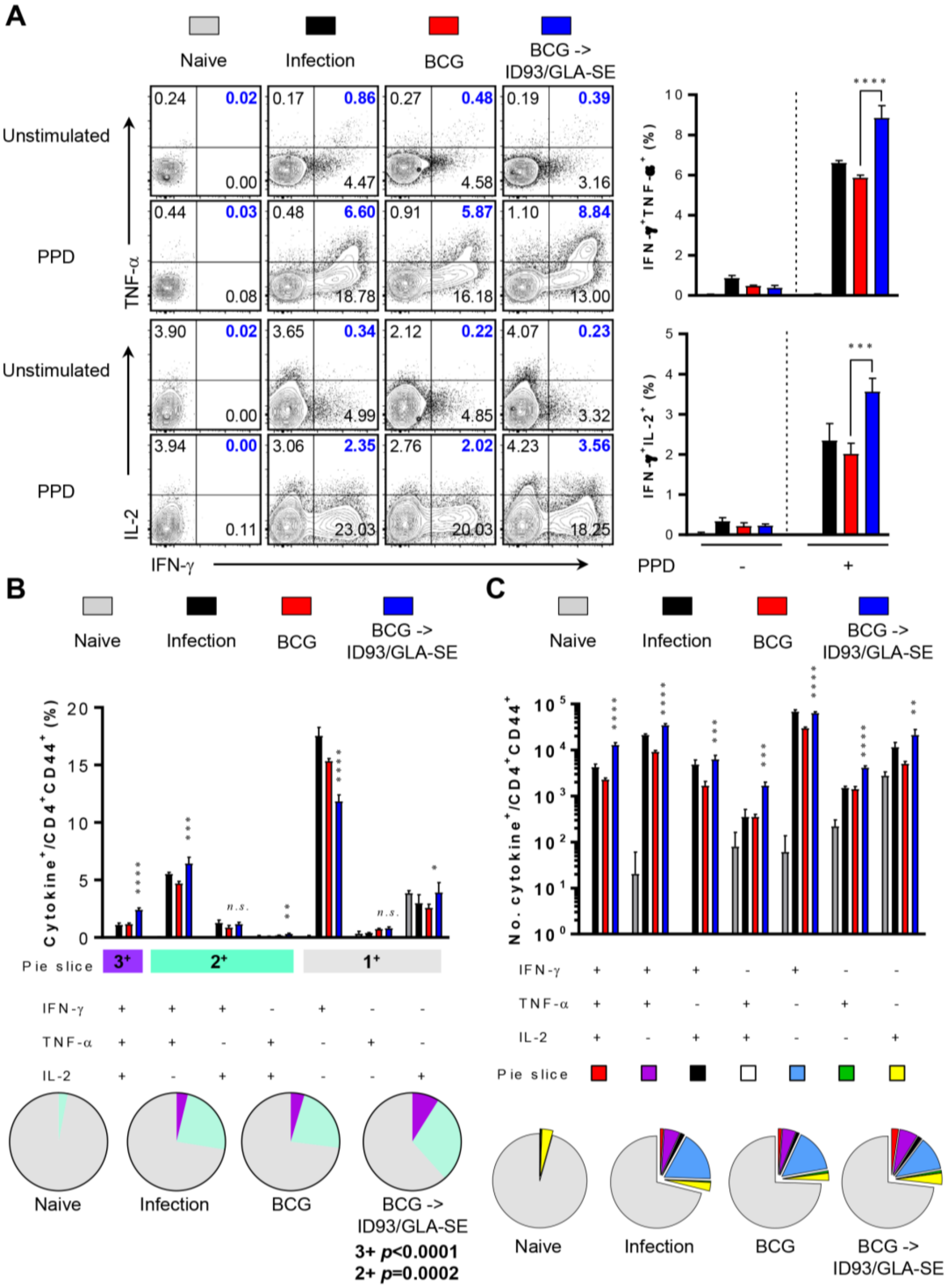

The mice in each group were sacrificed at 16 weeks following infection with Mtb strain K, and lung cells were treated with PPD (2 μg/ml) at 37°C for 12 hours in the presence of GolgiStop and GolgiPlug. (A) Upon stimulation with the PPD, the frequencies of Ag-specific $CD4^+CD44^+$ T-cells co-producing IFN-γ^+TNF-α^+ or IFN-γ^+IL-2^+ in the lung cells from each immunised group were evaluated using flow cytometry and described as representative dot plots and bar graphs. (B) PPD-stimulated lung cells from each immunised group were evaluated based on the percentage of total $CD4^+CD44^+$ T-cells with different patterns of cytokine production and described as bar graphs (S5B, upper). The pie charts summarise the fractions of triple (3^+, purple), double (2^+, light jade), and single (1^+, grey) $CD4^+CD44^+$ T-cell producers of IFN-γ, TNF-α and IL-2 in each immunised group (S5B, lower). (C) The actual number of PPD-specific polyfunctional T-cells among total $CD4^+CD44^+$ T-cells from each immunised group was represented as the bar graphs (S5C, upper). The mean number of cytokine-positive cells are displayed as pie charts with multiple colour fractions, and the number of the remaining cell population in the total $CD4^+CD44^+$ T-cells was described as grey fraction in the pie charts (S5C, lower). The experimental results are presented as the mean ± SD from 8 mice from each group. Statistically significant differences between the groups were determined using an unpaired Student's *t* test. *n.s.*; not significant, * $p<0.05$, ** $p<0.01$, *** $p<0.001$, and **** $p<0.0001$ comparing the BCG immunised mice and BCG-primed ID93/GLA-SE boosted mice.

Figure S6. Analysis of PPD-specific polyfunctional CD4+ T-cells in the spleens of BCG primed, ID93/GLA-SE boosted mice after infection with Mtb Beijing strain K.

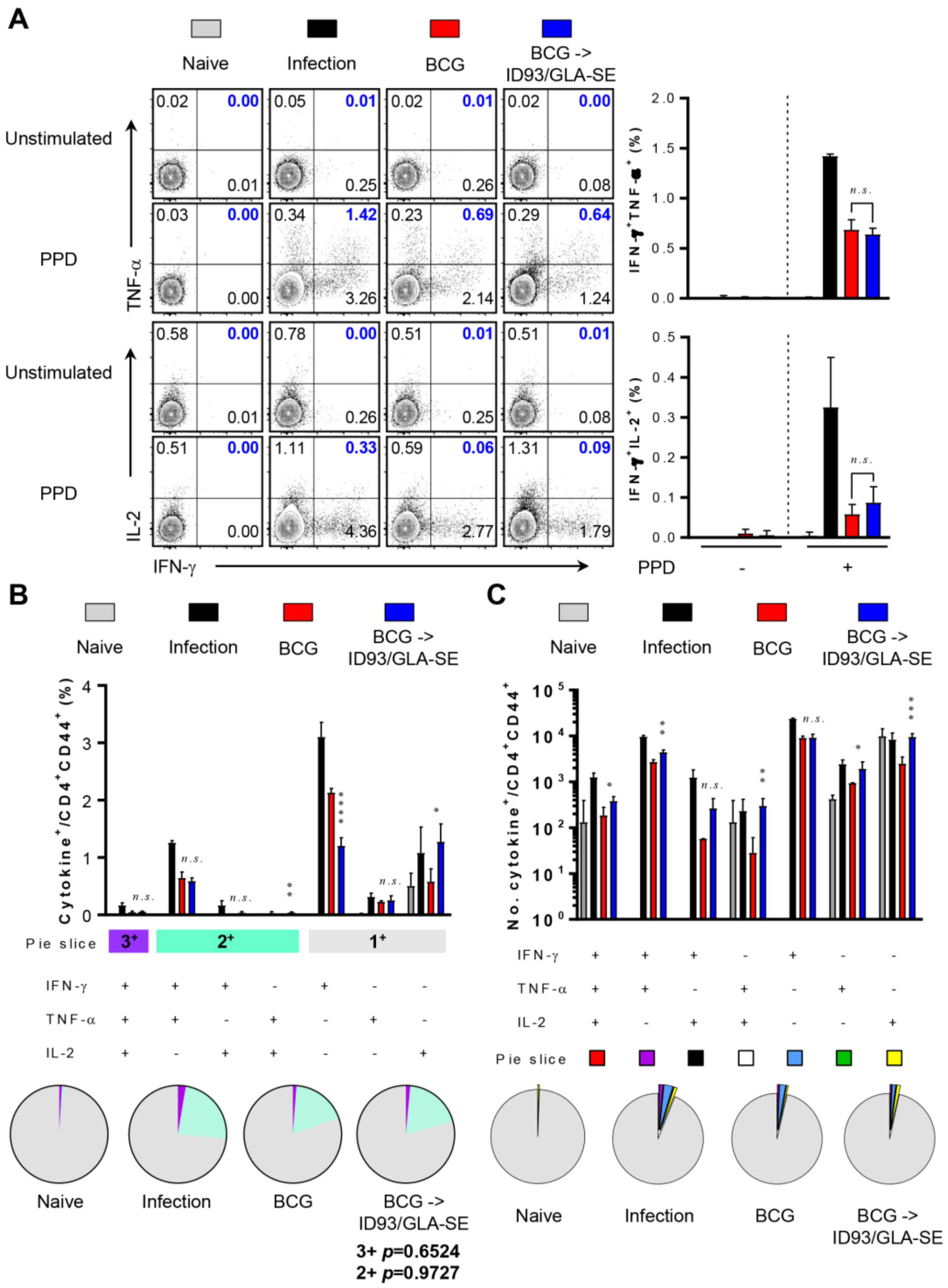

(A) Upon ex vivo stimulation of spleen cells with PPD, the frequencies of Ag-specific

$CD4^+CD44^+$ T-cells co-producing IFN-γ^+TNF-α^+ or IFN-γ^+IL-2^+ from each immunised group were evaluated using flow cytometry and described as representative dot plots and bar graphs. (B) PPD-stimulated spleen cells from each immunised group were evaluated based on the percentage of total $CD4^+CD44^+$ T-cells with different patterns of cytokine production and described as bar graphs (S6B, upper). The pie charts summarise the fractions of triple (3^+, purple), double (2^+, light jade), and single (1^+, grey) $CD4^+CD44^+$ T-cell producers of IFN-γ, TNF-α and IL-2 in each immunised group (S6B, lower). (C) The actual number of PPD-specific polyfunctional T-cells among total $CD4^+CD44^+$ T-cells from each immunised group was represented as the bar graphs (S6C, upper). The mean number of cytokine-positive cells is displayed as pie charts with multiple colour fractions, and the number of the remaining cell population in the total number of $CD4^+CD44^+$ T-cells was described as grey fraction in the pie charts (S6C, lower). The experimental results are presented as the mean ± SD from 8 mice per group. Statistically significant differences between the groups were determined using an unpaired Student's *t* test. *n.s.*; not significant, * $p<0.05$, ** $p<0.01$, *** $p<0.001$, and **** $p<0.0001$ comparing the BCG immunised mice and BCG-primed ID93/GLA-SE boosted mice.

Figure S7. Protective cytokine profiles in the spleen of ID93/GLA-SE boosted mice upon antigen re-stimulation at 16 weeks post-infection.

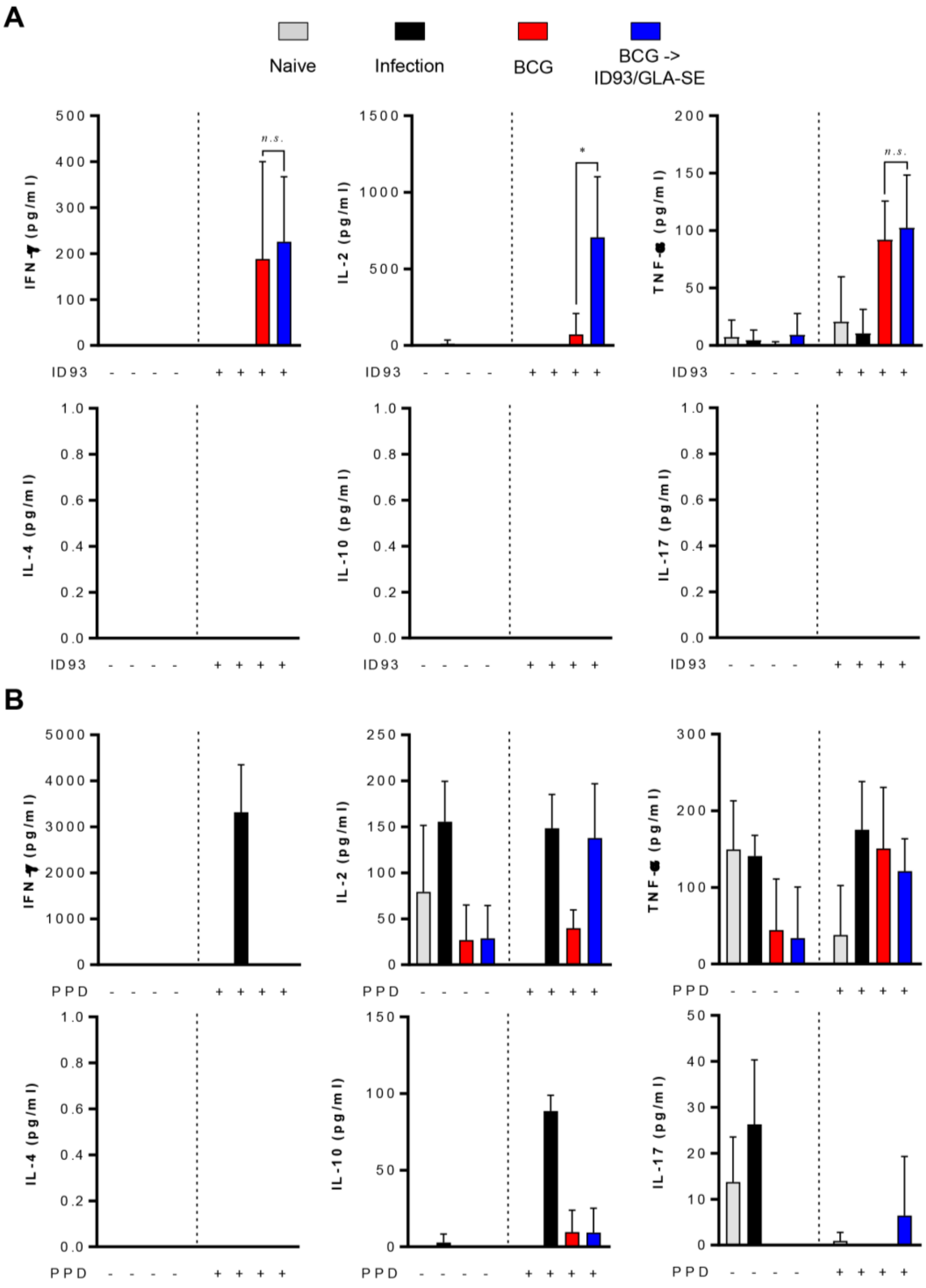

(A, B) Splenocytes from naïve, infected, BCG-immunised and BCG-primed ID93/GLA-SE

boosted mice (n = 4 mice/group at 16 weeks post-infection in each group) were stimulated with ID93 (1 μg/ml) or PPD (2 μg/ml) at 37°C for 12 hours, and the supernatant was collected and assayed for cytokines by ELISA. Statistically significant differences between the groups were determined using an unpaired Student's *t* test. *n.s.*; not significant, * $p<0.05$ comparing the BCG immunised mice and BCG-primed ID93/GLA-SE boosted mice.
